# Spleen-based proteogenomics reveals that *Escherichia coli* infection induces activation of phagosome maturation pathway in chicken

**DOI:** 10.1080/21505594.2022.2150453

**Published:** 2023-01-04

**Authors:** Jiahui Shi, Songhao Jiang, Qiang Wang, Jilin Dong, Huiming Zhu, Peijia Wang, Shuhong Meng, Zhenpeng Zhang, Lei Chang, Guibin Wang, Xiaoqin Xu, Ping Xu, Yao Zhang

**Affiliations:** a State Key Laboratory of Proteomics, Beijing Proteome Research Center, National Center for Protein Sciences Beijing, Research Unit of Proteomics & Research and Development of New Drug of Chinese Academy of Medical Sciences, Institute of Lifeomics, Beijing, China; b College of veterinary medicine, Yangzhou University, Yangzhou, China; c Department of Biomedicine, School of Medicine, Guizhou University, Guiyang, China; d Program of Environmental Toxicology, School of Public Health, China Medical University, Shenyang, China

**Keywords:** White Leghorn chicken, *Escherichia coli* O78, spleen, quantitative proteome, transcriptome

## Abstract

Avian pathogenic *Escherichia coli* (APEC) leads to economic losses in poultry industry and is also a threat to human health. Various strategies were used for searching virulence factors, while little is known about the mechanism by which APEC survives in host or is eliminated by host. Thus, chicken colibacillosis model was constructed by intraperitoneally injecting *E. coli* O78 in this study, then the protein dynamic expression of spleen was characterized at different post-infection times by quantitative proteome. Comparative analysis showed that *E. coli* induced significant dysregulation at 72 h post infection in spleen tissue. Transcriptomic method was further used to assess the changes of dysregulated proteins at 72 h post infection at the mRNA level. Total 278 protein groups (5.7%) and 2,443 genes (24.4%) were dysregulated, respectively. The upregulated proteins and genes were consistently enriched in phagosome and lysosome pathways, indicating *E. coli* infection activates phagosome maturation pathway. The matured phagolysosome might kill the invasive *E. coli*. This study illuminated the genetic dysregulation in chicken spleen at the protein and mRNA levels after *E. coli* infecting and identified candidate genes for host response to APEC infection.

## Introduction

Colibacillosis was caused by **A**vian **P**athogenic ***E**scherichia **c**oli* (APEC) infection in birds of all ages, which is an acute and mostly systemic disease resulting in huge economic losses to the poultry industry worldwide [1]. Furthermore, contaminated meat and eggs pose a threat to food safety and human health [2,3]. APEC constitutes a very diverse groups, among which O78 has been one of the most predominant serogroup [4,5]. Elucidating the chicken resistance mechanisms to APEC infection becomes urgent and important for developing sustainable strategies of effective vaccines and genetic selection of poultry for enhancing innate resistance [6].

Virulence factors responsible for bacterial invasion and pathogenesis have been the major focus in the study of APEC pathogenicity by genome [7], transcriptome [7], and proteome [8] on bacteria itself. In addition, host genomic [9] and transcriptomic [10] investigations have been performed for searching pathogenic genes and markers of APEC susceptibility or drug resistance. While there were few studies on the whole proteome of APEC infection in chicken tissues except sera sample [11,12].

*E. coli* invades the blood after infecting the respiratory tract, and then infects internal organs, such as liver and spleen, causing septicaemia and eventually death. The spleen is the main immune organ of chicken in response to infection and foreign antigens, which participates in humoral and cellular immune responses with its role in lymphocyte generation, maturation, and storage [13–15]. As the largest lymphoid organ of chicken, the spleen plays an important role in the immune system than that of mammal due to the maldevelopment of its lymph nodes and vessels [14,16]. Splenic enlargement is one of the typical characteristics after *E. coli* infection or inoculation. Gene expression of the chicken spleen is commonly used as an indicator of immune response [17,18], while spleen responsive and resistant mechanisms have not been systematically studied at the protein level.

In this study, we successfully constructed systemic infection model of the White Leghorn chicken with O78 strain, and applied quantitative proteome and transcriptome to investigate the dynamic changes in spleen tissue at the protein and mRNA levels after *E. coli* infection. Comparative study of infection characteristics and proteome at various time points showed that 72 h was an important infection time for spleen tissue, in which *E. coli* induced consistently significant dysregulation in phagosome pathway. This repertoire of deep and accurate proteomics and genomics provides the framework to understand the host resistance mechanism against APEC infection.

## Materials and methods

### Ethics statements

All animal experiments were complied with the Animal Welfare Committee of Institutes of Animal Sciences, Chinese Academy of Agricultural Sciences (IASCAAS), and approved by the Administrative Committee for Laboratory Animals (Permission number: SCXK-2014–0005).

### Bacterial culture

*E. coli* O78 (CVCC1418) was obtained from China Institute of Veterinary Drugs Control and was grown at 37 °C in Luria-Bertani (LB) broth (Shanghai Zhongke Insect Biotechnology Development Co., Ltd, Shanghai, China) in a shaker until an optical density at 600 nm (OD_600_) reached about 0.6. *E. coli* cells were collected by centrifugation and washed by sterile saline before inoculations.

### Chicken infection

All healthy one-day-old, specific pathogen-free (SPF), White Leghorn chickens, were acclimated for 15 days before infection. The chickens from the infection group were intraperitoneally inoculated with 3 × 10^8^ colony forming units (CFU) of *E. coli* O78 resuspended in 0.5 mL of sterile saline, and the chickens from the control group were inoculated with the same volume of sterile saline as the sham control. All chickens were numbered and fitted with exclusive leg tags.

**Figure 1.**
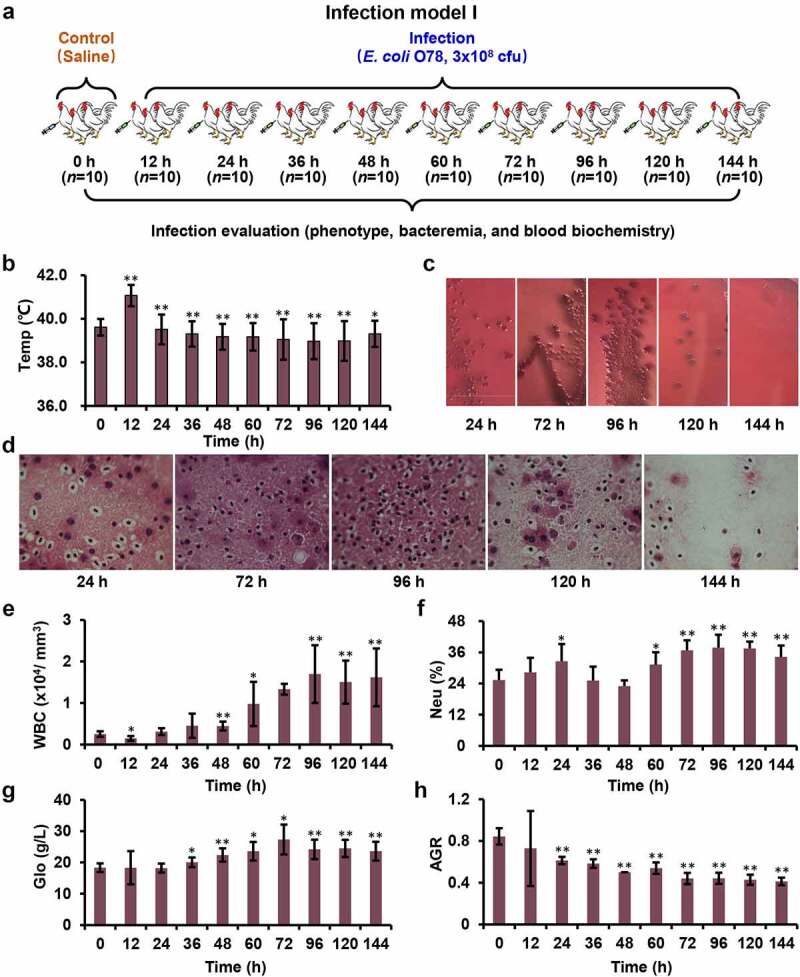
Systemic model of APEC infection. (a) Model I was designed to evaluate the effects of infection at different time points. (b) Chickens showed obvious fever after infection (magnification of 100×). (c) The blood samples coated on the MacConkey plates at different time points. (d) The sections from liver tissue stained with haematoxylin and eosin. (e-h) The complete blood cell count for blood sample at different time points.

**Figure 2.**
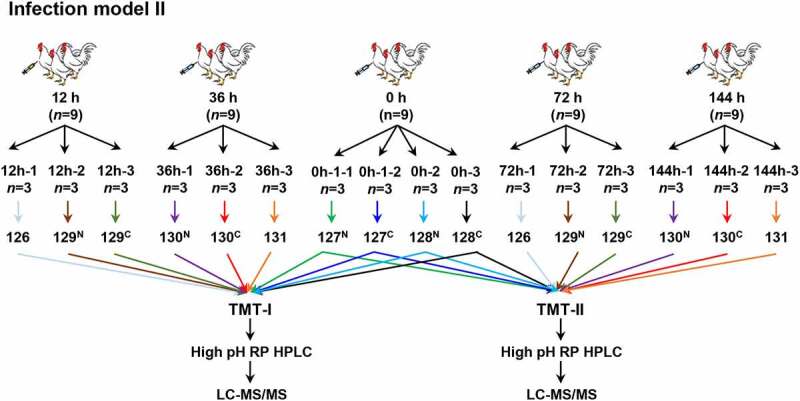
Flowchart of the spleen-based proteomic study. Infection model II was carried out to obtain the samples at 12 h, 36 h, 72 h and 144 h for quantitative proteome by TMT 10-plex labeling.

This model construction included two separate experiments (Infection model I and II). Model I was designed to evaluate the effects of infection at different time points after inoculation with *E. coli* O78. One hundred chickens were randomly divided into 10 groups, 10 chickens in each group, including control group (0 h), and infecting groups (12 h, 24 h, 36 h, 48 h, 60 h, 72 h, 96 h, 120 h, and 144 h) after weighing (Figure 1a). All the surviving chickens from these 10 groups were monitored for general body condition, anorexia, depression, morbidity, mortality, and clinical symptoms including blood count, blood chemistries, etc. On the basis of the results of model I, model II was carried out to study the tissue responding mechanism at different infection time (12 h, 36 h, 72 h, and 144 h). Nine chickens of each time point were prepared for proteomic (Figure 2) and transcriptomic experiments.

### Sample collection

Total 2-mL blood samples were obtained from all surviving chickens in model I through jugular vein by a sterilized syringe. Among them, 1.5 mL sample was collected in a tube without heparinization for biochemistry index analysis, and 0.5 mL sample was collected in an anticoagulant tube for hemocyte analysis. Each 1.5 mL blood sample was centrifuged at 4,000 rpm for 10 min to recover serum before analyzing at the Disease Detection Center of Yangzhou University Animal Hospital. And 0.45 mL blood sample was sent for complete blood cell count. The remaining 50 μL blood was plated onto a plate containing MacConkey medium (Haibo Corp., Qingdao, China) to culture *E. coli* O78 by incubating at 37°C for 18 h. The colonies were counted to evaluate the states of bacteremia of these samples.

### Post-mortem examination

Post-mortem examinations were performed immediately after blood sampling or the death of chickens. Seven organs of the surviving chickens were aseptically removed in model I for pathological observation and comparison, including heart, liver, spleen, lung, kidney, intestine, and bursa. The livers of the dead chickens were also obtained for rigid contact lens staining observation. The spleens of the surviving chickens in model II were aseptically sampled, weighed, sliced, and washed with PBS buffer before frozen in liquid nitrogen.

### Histopathologic examination

The liver tissues were stained with hematoxylin and eosin. The stained image was checked under a Leica microscope MC15 (Leica, Wetzlar, Germany) and analyzed with Olympus cellSens Dimensions (version 1.7) software (Olympus, Tokyo, Japan).

### Proteome sample preparation

Each spleen tissue sample (0.2 g) from model II were ground in liquid nitrogen and sonicated in lysis buffer [8 M urea, 50 mM iodoacetamide (IAA), 100 mM NaH_2_PO_4_, 10 mM Tris-HCl, 1× protease inhibitor cocktail (Roche, Basel, Switzerland)] on ice for 10 min as follows: 2 s at 30% W and then a 4 s pause. The total cell lysate was obtained by centrifugation of 13,300 ×g at 4°C for 15 min and measured the concentration by a gel-assisted strategy, as described previously [19]. To reduce the individual variation, 9 protein samples of each time point were randomly combined into 3 groups (*n* = 3/group). The biological duplicates were used to determine the filtering criteria [20]. 100 μg proteins of each group were reduced with 5 mM DTT, alkylated with 10 mM IAA, and separated by a short 10% SDS-PAGE (0.7 cm). The proteins were digested in-gel with a final concentration of 12.5 ng/μL trypsin at 37°C for 14 h [21,22].

The extracted peptides were labeled with 10-plex TMT reagent (Thermo Fisher Scientific, Rockford, IL, USA). Two TMT-labeling groups, TMT-I and TMT-II, were performed. In TMT-I, 10 samples, 0 h-1–1, 0 h-1–2, 0 h-2, 0 h-3, 12 h-1, 12 h-2, 12 h-3, 36 h-1, 36 h-2, and 36 h-3 were labeled with 127^N^, 127^C^, 128^N^, 128^C^, 126, 129^N^, 129^C^, 130^N^, 130^C^, and 131, respectively. In TMT- II, 10 samples, 0 h-1–1, 0 h-1–2, 0 h-2, 0 h-3, 72 h-1, 72 h-2, 72 h-3, 144 h-1, 144 h-2, and 144 h-3 were labeled by 127^N^, 127^C^, 128^N^, 128^C^, 126, 129^N^, 129^C^, 130^N^, 130^C^, and 131, respectively. The labeled peptides were mixed after quality control.

### Peptide fractionation and LC-MS/MS analysis

The mixed TMT-labeled peptide sample was dried and dissolved with 100 μL buffer A [98% dd H_2_O, 2% acetonitrile (ACN), pH = 10], and fractionated using a high pH reverse phase HPLC system (L-3120; Rigol, Beijing, China) with an increasing gradient of buffer B (2% dd H_2_O and 98% ACN, pH = 10) as described [23]. Each fraction was dried and suspended in loading buffer [0.1% formic acid (FA) and 1% ACN] for LC-MS analysis.

The peptides were separated and analyzed by an EASY-nanoLC1200 (Thermo Fisher Scientific, Waltham, MA, USA) equipped with a Q Exactive HF (Thermo Fisher Scientific) as described. Briefly, the samples (500 ng) were loaded onto a self-packed capillary column (150 μm i.d. ×15 cm, 1.9 μm C_18_) with a 135 min gradient: 5–10% D for 20 min, 10–28% D for 80 min, 28–42% D for 20 min, 42–95% D for 3 min, and 95% D for 12 min (Buffer C: 0.1% FA in H_2_O; Buffer D: 0.1% FA and 80% ACN; flow rate: 300 nL/min). MS scans were performed at a resolution of 120,000 over a mass range between 375 and 1400 *m/z*. For MS/MS scan, the 15 most intense ions with charge state 2 to 6 were subjected to fragmentation via high energy collision-induced dissociation (HCD). For each scan, 100,000 ions were accumulated over a maximum allowed fill time of 100 ms. Exclusion of precursor ion masses over a time window of 30 s was used to reduce repeated peak fragmentation.

### MS/MS data analysis

The MS/MS raw data were searched by MaxQuant (v1.6.17.0) against the *Gallus gallus* reviewed database (18,124 entries, 19 June 2019), in which the *E. coli* database (4,391 entries, 23 June 2019) was downloaded from UniProt (https://www.uniprot.org/) and 245 common contaminant database. Fully tryptic peptides with as many as 2 missed cleavages were allowed. Cysteine carbamidomethyl and TMT 10-plex (N-term/K) were set as fixed modification, while oxidation of methionine was set as variable modification. The tolerance of the precursor and fragment ions was set to 20 ppm. The minimum length of peptides was set to more than 7 amino acid (AA). The peptides and proteins were filtered based on 1% false discovery rate (FDR).

### RNA library construction and sequencing

RNA-sequencing was performed for further evaluating the gene changes at 72 h post infection. To avoid the influence of individual and technical variation, 9 biological samples of two groups (control and 72 h post infection) and 2 technical repetitions were considered. Total RNA was extracted and purified from spleen tissues using the TRIzol reagent (Invitrogen, Carlsbad, CA, USA). RNA purity was detected by the NanoPhotometer spectrophotometer (Implen GmbH, München, Germany). RNA integrity was measured on the Agilent 2100 bioanalyzer (Agilent Technologies, CA, USA). High-quality RNA was enriched by Sera-Mag oligo (dT) beads (Thermo Scientific, Indianapolis, IN, USA) and fragmented with the NEB Fragmentation kit (NEB, Ipswich, MA, USA). Sequencing libraries were constructed according to the protocol of NEBNext® UltraTM RNA Library Prep Kit for Illumina (NEB, Ipswich, MA, USA). Library concentration was quantified by the Qubit2.0 Fluorometer (Invitrogen, Carlsbad, CA, USA), and quality was measured on the Agilent 2100 bioanalyzer and read length was 150 bp. In order to ensure the quality and reliability of RNA-sequencing data, we removed the reads with adapters and the reads containing N, and removed the low-quality reads (base number of Qphred ≤20 accounting for more than 50% of the whole read length). The clean data were aligned to the *G. gallus* genome (Gallus_gallus_ncbi_GCF_000002315.6_GRCg6a) using Hisat2 (v2.1.0) [24]. After filtering, 11.82 ± 0.99 GB clean bases of each sample were generated and aligned. The FPKM (fragments per kilobase of exon model per million) was counted and calculated to estimate the expression levels of genes in each sample.

### RNA extraction and qRT-PCR

Total RNA of spleen tissues at different infecting times were isolated with TRIzol reagent (Invitrogen, Carlsbad, CA, USA) according to the instructions. cDNA was obtained by ReverTra Ace® qPCR RT Master Mix (TOYOBO, Osaka, Japan). β-actin was used as the endogenous control, the mRNA levels of representative genes in phagosome maturation were determined via qRT-PCR. The primer sequences for amplifying the phagosome maturation-related genes are listed in Table S15. The qRT-PCR was performed by SYBR green realtime PCR Master Mix (TOYOBO, Osaka, Japan). Differences in the mRNA expression of genes were compared using unpaired two-samples Student’s *t*-test. All statistical analyses were performed using R software (4.0.2). *p-*value of less than 0.05 was considered statistically significant. The fold change was calculated based on the mean of infection group against each control group (*n* = 6).

### Bioinformatic analysis

Biological or technical variability was evaluated by the intensity ratio of the control and infection groups based on their correlation coefficient and standard deviation (SD). Gene or protein ratios between infection and control >3×SD and *p-*value <0.05 were considered as regulated differentially expressed genes (DEGs) or proteins (DEPs). The total proteomic quantification datasets were median-normalized, and *p-*value was calculated by Perseus (v1.6.6.0).

The DAVID online platform (https://david.ncifcrf.gov/) [25] was used for functional analysis of differentially expressed proteins and genes.

### Data availability

The MS/MS raw data has been deposited to the iProX partner repository [26] with the identifier IPX0003381002.

The transcriptome data in this study have been deposited in NCBI’s Gene Expression Omnibus (GEO) with the accession number GSE182967. The following secure token has been created to allow review of record GSE182967: uhcnkuearfabzqn.

## Results

### E. coli O78 causes avian systemic infection

Avian systemic infection model was successfully constructed by intraperitoneal inoculation of 3 × 10^8^ CFU of *E. coli* O78 cells into White Leghorn chickens. To assess the severity of infection, we compared the general and clinical symptoms at different post-infection time (Figure 1 and Table S1). At 12 h post infection, all chickens showed obvious fever symptoms (Figure 1b) with 0.5 to 1 degree of increasing temperature (Table S2). In contrast to 12 h, the respiratory symptom turned from light breathing to heavy breathing at 24–36 h post infection. The major systemic symptoms were flock together, chilliness with fever, deep voice, mental fatigue, extreme drowsiness, anorexia, diarrhea, ruffled feathers, closed eyes, as well as no reaction to acoustics, etc. The chickens with these symptoms account for more than 90% of the survivors. More recumbent chickens were observed at 48–72 h post infection, which exhibit opisthotonos, head tilt, and eyes closed, which account for more than 85% of the survivors. After 96 h post infection, these symptoms began to diminish or even disappear.

### E. coli infection caused serious tissue lesions at 72 h post infection

At necropsy, typical colibacillosis lesions were only observed in the infected group as described previously [27,28]. With persistent infection, the degree of tissue lesions increased gradually, including perihepatitis, pericarditis, splenectasis, and hypernephrotrophy. When the infection time extended to 60 h, perihepatitis and pericarditis were observed in all infected chickens (Figure S1). From 72 h post inoculation, splenectasis and hypernephrotrophy were detected in nearly half of the infected chickens. Splenectasis further suggested the immune and inflammatory response are evoked after *E. coli* infection, especially at 72 h post infection.

To determine bacterial dissemination, the blood samples collected at different time points were coated on the MacConkey plates (Figure 1c). Both numbers of bacterial clones cultured from 72 h and 96 h blood samples were significantly higher than that from the other time points. The CFU values in the liver tissues were observed as well (Figure 1d). Consistently, the number of bacterial CFU from spread liver was continuously increased from 24 to 96 h post infection. However, the CFUs were significantly reduced after 96 h.

### E. coli infection triggered stronger immune response at 72 h post infection

To further validate the clinical symptoms of avian systemic infection model, we checked the serum samples from these infected chickens. White blood cell (WBC) proliferation is a common feature of infectious diseases, especially in bacterial infections [29]. The WBC results did show gradually increase in the early stage of infection (after 12–72 h post infection), and stabilized after 96 h post infection (Figure 1e). Among WBCs, neutrophils (Neu) and lymphocytes (Lym) are the main functional cell groups to fight against infection. The number of Neu in serum increased from 12 to 24 h, remained constant at 36 h, slightly decreased at 48 h, significantly increased from 60 to 96 h, and gradually stabilized after 96 h post infection, respectively (Figure 1f). However, the Lym had no visible changes from 12 to 48 h post infection, while slightly decreased after 60 h post infection (Figure S2).

As an indicator of host resistance to bacterial infection, globulin (Glo) has been widely used for blood biochemical analysis item in clinic. The elevated globulin [30] has been used as one of the critical markers associated with inflammation and infection. After infection, the globulin increased gradually from 12 h post infection, peaked at 72 h, then decreased and remained essentially stable from 96 h to 144 h (Figure 1g & Table S4). The elevated globulin level in the whole process indicated the inflammation and infection of our chicken model. The decrease of albumin (Alb) to globulin ratio (AGR) [31,32], a simple blood parameter, has also been confirmed as a prognostic factor for various disease [33]. On our blood samples, AGR decreased gradually but significantly from 12 to 48 h post infection, however, with unknown reasons, increased slightly at 60 h and then continuously decreased to 72 h and remained stable (Figure 1h). The coincidence of 72 h time point of serious tissue lesions with the time of globulin and AGR significant fluctuation highlighted the key node of the subsequent omics studies.

In short, all of the symptoms, clinical anatomy, hematology, and biochemical indicators indicated that we have successfully constructed a chicken infection model with pathogenic *E. coli* that can meet the needs of multi-omics analysis. After 72 h of infection, the inoculated bacteria were sufficiently grown and fight with host *in vivo*, which was suitable for omics analysis.

### E. coli infection induced significant splenectasis symptom

In model II, we also found the spleen weight increased significantly with persistent infection times (*p-*value <0.001, Figure S3a & c). Similar spleen enlargement also observed in infecting groups (Figure S3b & d). As spleen is the main organ to fight against bacterial infection, the enlarged spleens at 12 h, 36 h, 72 h, and 144 h post infection were selected for further quantitative proteomics study (Figure 2).

### Proteomic study revealed E. coli infection caused various protein dysregulation in spleen tissue at different infecting time points

**Figure 3.**
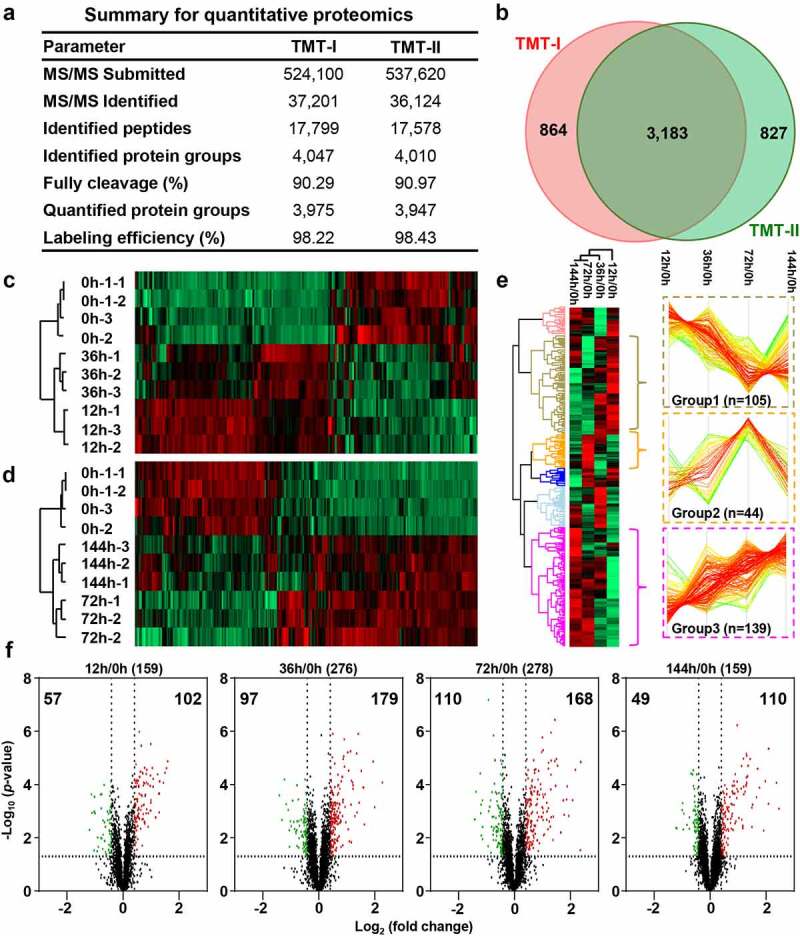
Dysregulated proteins of chicken spleens after *E. coli* infection. (a) The identified and quantified proteins from quantitative proteomics. (b). Venn diagram of protein groups identified from TMT-I and TMT-II. The heat map of dysregulated proteins from the (c) TMT-I (0 h/12 h/36 h) and (d) TMT-II (0 h/72 h/144 h). (e) The cluster proteins with persistent dysregulation at different time points after infection. (f) The volcano plot of dysregulated proteins between 12 h and 0 h, 36 h and 0 h, 72 h and 0 h, and 144 h and 0 h (log_2_ fold change >3×SD, *p*-value <0.05).

To understand the molecular mechanism underlying *E. coli* infection-induced chicken colibacillosis, the proteins from the control and *E. coli* infected chicken spleen tissues were compared using the 10-plex TMT method (Figure 2, Figure S4 & S5). In TMT-I, we identified 17,799 peptides corresponding to 4,047 protein groups (Figure 3a), of which 3,975 protein groups were simultaneously quantified by all 10 tags. In TMT-II, we detected 17,578 peptides corresponding to 4,010 protein groups (Figure 3a), of which 3,947 protein groups were quantified. The protein identification in this study was close to Ma *et al*. dataset of heat stress induction [34] (Figure S6). The labeling efficiency was more than 98% (Figure 3a). Total 3,183 protein groups were quantified in two TMT-labeling experiments, implying these two TMT labeling experiments had high reproducibility (Figure S7) and can be used for comparing analysis of protein expression at different infecting time points (Figure 3b).

The corresponding R^2^ values of two technical duplicates were >0.99 (Figure S8a & c). The mean values of the histograms of the technical duplicates from the two TMT 10-plex labeling samples were −0.148 and 0.145, and the SD values were 0.136 and 0.135, respectively (Figure S8b & d). These results indicate that the high reproducibility of the proteomic experiments is well controlled. Utilizing 3-fold of SD value (fold change >1.33 or <0.75) and *p-*value < 0.05 as filtering criteria (Figure 3f), DEPs were selected at 4 infecting times, including 159 DEPs at 12 h (Table S7–1), 276 DEPs at 36 h (Table S7–2), 278 DEPs at 72 h (Table S7–3) and 159 DEPs at 144 h (Table S7–4). The expression of these DEPs showed the consistent expression in repeat experiments (Figure 3c & d).

The principal component analysis (PCA) showed obvious difference between the control and different infected times (Figure S8e & f), implying various proteins were dysregulated after different infecting times.

### Diverse dysregulation models were caused at different infection times

Quantitative comparison found that DEPs from four time points were mainly clustered into three groups based on their protein expression (Figure 3e). The expression of the first group of 105 DEPs was continuously decreased from 12 h to 72 h, reached the lowest at 72 h, and increased at 144 h. These proteins were significantly enriched in NOD-like receptor signaling and influenza A pathway (Figure S9 & Table S8). The second group of 44 DEPs were up regulated from 12 h to 72 h, reached the highest expression at 72 h, but basically recovered at 144 h. These proteins were significantly enriched in ribosome, DNA replication, and cell cycle pathway (Figure S9 & Table S8). The third group of 139 DEPs were rapidly up-regulated from 12 h to 72 h, but remained constant at 144 h, and they were enriched in the protein processing in endoplasmic reticulum, protein export, and phagosome pathway (Figure S9 & Table S8).

The DEPs at different time points showed that the number and dispersion of differentially expressed proteins in the spleen kept aggravated from 12 h to 72 h, and fell back at 144 h, which indicated that the symptoms of colibacillosis began to reduce, and the infected chickens gradually recovered from 72 h to 144 h. These results also indicated that 72 h after infection is the key time point for the gradual reduction of colibacillosis symptoms after reaching the peak.

### Gene ontology analysis showed E. coli infection induces a strong immune response at 72 h

Gene ontology (GO) analyses [25] of the 278 DEPs at 72 h showed that *E. coli* infection induced a strong immune response. Among them, the up-regulated proteins (168) were mainly enriched in complement activation (classical pathway), positive regulation of B cell activation, phagocytosis, recognition, engulfment, defense response to bacterium, B cell receptor signaling pathway, innate immune response, cytolysis and immune response, etc. The down-regulated proteins (110) were significantly enriched in actin filament-based movement, triglyceride metabolic process, lipid storage, actin polymerization or depolymerization, regulation of cytosolic calcium ion concentration, positive regulation of cell adhesion molecule production, etc. (Figure S10).

### Parallel transcriptomic analysis verified the expression of DEPs

Both model construction and quantitative proteomic experiment found that 72 h post infection was the most severe time point. Moreover, the DEPs from 72 h were significantly enriched in the immune related pathways such as complement activation, B cell activation, phagocytosis, indicating that *E. coli* infection caused a strong immune response of host at this time point.

**Figure 4.**
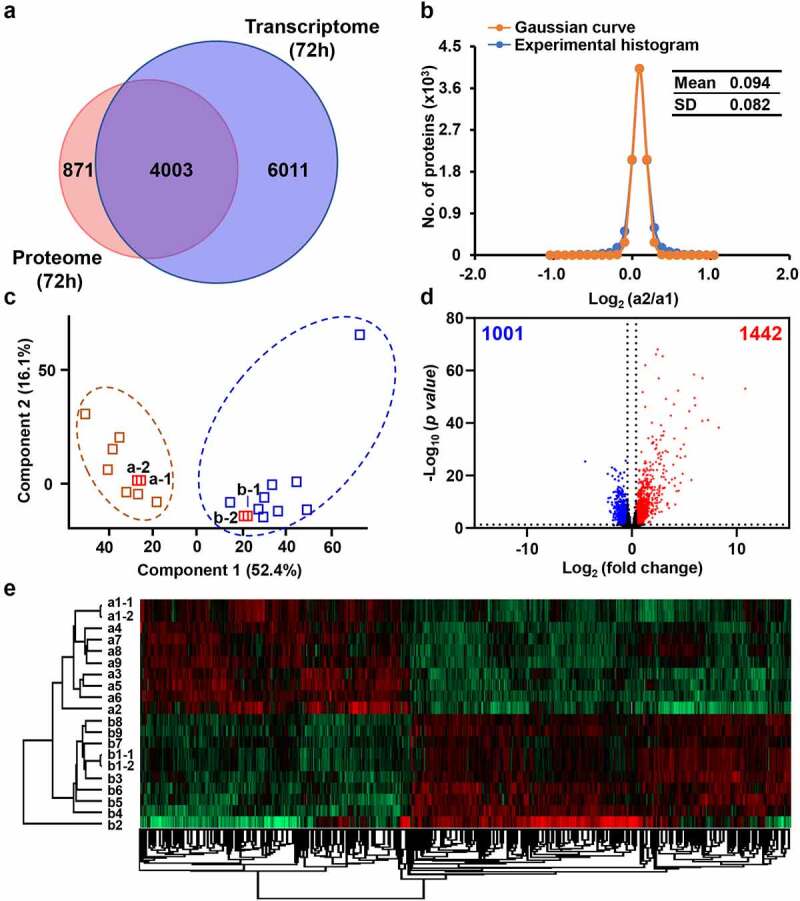
Dysregulated genes of chicken spleens at mRNA level after *E. coli* infection. (a) Overlap of identified genes between proteome and transcriptome analysis. (b) The Gaussian fitting curve of log_2_ ratio of the FPKM of technical replicates a1–1 and a1–2. (c) The PCA analysis between the control and infected chickens. (d) The volcano plot of 1,442 up-regulated genes and 1,001 down-regulated genes (log_2_ fold change >5xSD, *p-*value <0.05). (e) The heat map comparison of dysregulated genes between the control and infected chickens.

Therefore, we further selected 72 h chicken spleen samples for RNA sequencing to verify the findings in the above quantitative proteomics. In transcriptome (Figure S11 & Table S9), a total of 10,014 genes were identified. Among them, 4,003 were synchronously detected at both the mRNA and protein levels (Figure 4a). Both corresponding R^2^ values for technical repeats of control and infected samples were 1.00 (Figure S12a & c). The mean values of the histograms of biological duplicates of control and infected samples were 0.094 and 0.093, and the SD values were 0.082 and 0.083, respectively (Figure 4b & S12b). These results indicated the high quality of our transcriptomic data as well.

### An elicited immune response from the spleen transcriptomic level

The PCA showed obvious difference and separation of control and infected chickens (Figure 4c). Utilizing 5-fold of SD value (fold change >1.33 or <0.75) and *p-*value < 0.05 as filtering criteria, 2,443 genes were selected as DEGs upon *E. coli* infection. Among them, 1,442 genes were upregulated and 1,001 genes were downregulated, respectively (Figure 4d & Table S10).

The heat map showed that the upregulated and downregulated genes were clearly clustered in two groups (Figure 4e). The DEGs substantially outnumber DEPs. Among them, 127 were overlapped, occupied 45.7% of DEPs and 5.2% of DEGs (Figure S13 & Table S11).

GO analyses showed that the upregulated DEGs (1,442) were significantly enriched in inflammatory response, cytokine-mediated signaling pathway, cellular response to tumor necrosis factor, defense response to virus, cellular response to lipopolysaccharide, immune response, monocyte chemotaxis, neutrophil chemotaxis, and cellular response to interleukin-1, etc. The downregulated genes (1,001) were mainly clustered in translation, cytoplasmic translation, stabilization of membrane potential, calcium-mediated signaling, positive regulation of vascular endothelial growth factor signaling pathway and positive regulation of signal transduction by p53 class mediator, etc. (Figure S14). The enrichment of immune response-related genes from the up-regulated genes is heavily consistent with what we observed in proteomic study.

## Discussion

**Figure 5.**
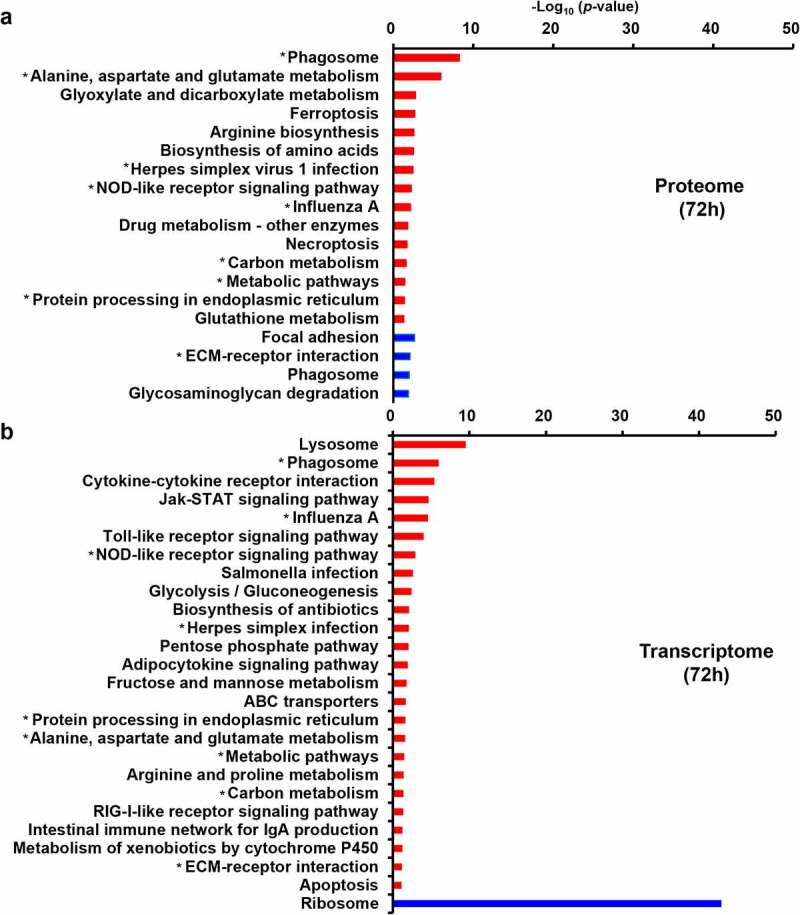
KEGG pathway analysis. (a) KEGG pathway analysis of the dysregulated DEPs from quantitative proteomics. (b) KEGG pathway analysis of the dysregulated DEGs from transcriptomic. The red and blue represent up-regulated and down-regulated genes, respectively. The asterisk represents pathways in which both dysregulated DEPs and DEGs are enriched.

**Figure 6.**
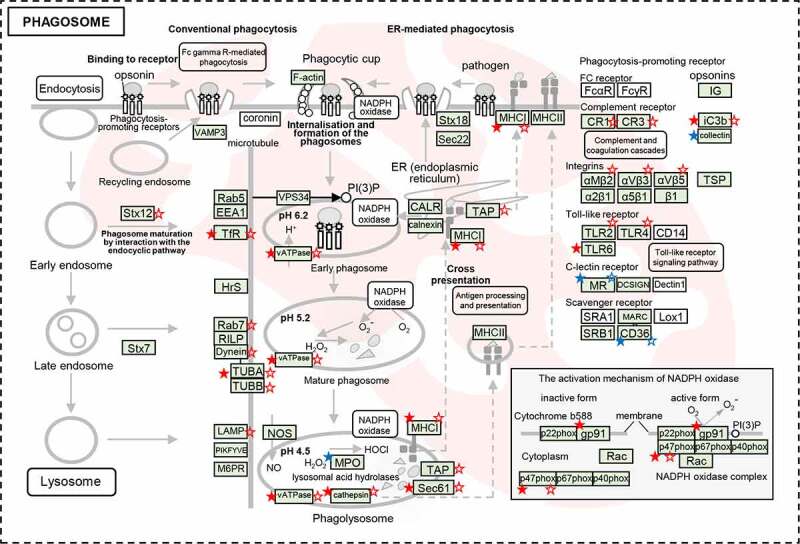
The dysregulated DEPs and DEGs were significantly enriched in the pathway of phagosome maturation. Schematic model for phagosome maturation after *E. coli* infection. The significantly dysregulated genes at protein level and mRNA level were marked by stars (solid stars: protein level dysregulation, hollow stars: mRNA level dysregulation, red: up-regulation, blue: down-regulation).

Based on the quantitative proteomic and transcriptomic data, KEGG pathway analysis revealed that the upregulated DEPs and DEGs were concurrently enriched phagosome, alanine, aspartate, and glutamate metabolism, herpes simplex virus 1 infection, NOD-like receptor signaling pathway, influenza A, carbon metabolism, metabolic pathways, protein processing in endoplasmic reticulum and ECM-receptor interaction (Figure 5 & S15, Table S12 & S13). Interestingly, phagosome related genes were most significantly enriched at both of the mRNA and protein levels (Figure 6 & Table S14), which participate a series of phagosome maturation steps. Of these, the members of vacuolar ATPase (vATPase) family recruiting to phagosomes [35,36] at early stage, were heavily enriched by upregulated genes, including ATP6V1A, ATP6V1B2, ATP6V1E1, ATP6V1G1, ATP6V0D1, and ATP6V1 H. The GTPases of the Rab family, representing an important protein group involved in phagosome maturation [37], including the mature phagosome Rab7 were upregulated on RNA levels (RAB7A, 1.66). The up-regulation of Rab7 [38] represents the transition of late phagosome (Figure S16).

According to the fold changes of the DEPs at different time points, we observed that the upregulated proteins were consistently clustered in the phagosome pathway, gradually increased from 12 h to 72 h, but decreased to a certain extent at 144 h. Similar phenomena also existed in the 6 downregulated proteins. After reaching the lowest point at 72 h, the protein abundance would slightly rise at 144 h (Figure S16b). These results were consistent with the data of the blood cell count and the dysregulated proteins at different infecting time points, indicating that about 72 h is the most serious period for poultry after infection with *E. coli*.

Finally, the late phagosome interacts with lysosomes to develop into a phagolysosome. LAMP1, is required for phagolysosomal fusion, which upregulated at the mRNA (2.07). During phagosome maturation, its degradative capacity and antimicrobial activity is augmented by the acquisition of hydrolytic enzymes and the production of oxygen radicals by NADPH oxidases (NOX2). Total 43 DEGs were largely enriched in lysosome pathway (Figure S14b). In particular, the genes related to lysosomal acid hydrolases proteases were upregulated, including proteases [cathepsin (CTSB, CTSC, CTSD, CTSH, and CTSS) and LGMN], glycosidases (GLA, GAA, GUSB, HEXA/B, and MANB), sulfatases (ARS and SGSH), lipases (LIPA), nuclease (DNase II), sphingomye linase (SMPD1), and ceramidase (ASAH1). NOX2 controls phagosomal pH by activating NOX2 (CYBB), such as NCF1C (p47*phox*) to the membrane subunits (Figure 6 & Table S14). NCF1C was upregulated at both of the mRNA (1.64) and protein (1.76) levels. These results implied that hydrolases proteases and NOX2 played roles in forming an increasingly acidified compartment for effectively eliminating the invasive *E. coli*.

In addition, various factors induced by pathogen infection were identified that have direct or indirect influence on phagosome maturation. Pathogens that are recognized by pattern recognition receptors (PRRs). The phagocytosis-promoting receptors were upregulated after *E. coli* infection, including complement receptor (CR1, CR3, and iC3b), integrins (αMβ2, αVβ3, and αVβ5), toll-like receptor (TLR2, TLR4, and TLR6), might accelerate the phagosome maturation (Figure 6).

Further qRT-PCR assays showed that representative genes of phagosome maturation were significantly upregulated in response to *E. coli* infection including ATP6V1A, ATP6V1B2, BF2, CTSS, and TFRC, while COLEC12 was downregulated at 72 h (Figure S17 and Table S16). In addition, these representative genes of phagosome maturation were dysregulated at different infecting time points.

These results revealed that *E. coli* infection induced phagosome maturation in spleen. The matured phagosomes become phagolysosome with acidic, hydrolytic, and oxidative lumen, which could effectively kill and digest the phagocytized microbes [39].

## Conclusion

In summary, APEC infection model was successfully constructed using *E. coli* O78 in White Leghorn chickens with typical general and clinical symptoms. To further investigate the immune response, quantitative proteomics and transcriptomics were performed to compare the gene expression of chicken spleen between the control and infected groups at different time points. Quantitative comparison showed that *E. coli* infection significantly disturbed the gene expression both at the protein and mRNA levels. Comparing analysis of the symptoms in model construction and the dysregulated proteins in proteomic study showed that 72 h was the most severe period for avian colibacillosis. The DEPs and DEGs were consistently and notably enriched in phagosome pathway and took part in phagosome maturation. A number of molecules, including GTPases, vATPase, and membrane fusion components were upregulated, which were recruited to phagosomal surfaces and drive phagosome maturation. This study revealed that phagosome maturation was activated for *E. coli* clearance in spleen.

## Supplementary Material

Supplemental Material
