# Supplementary material for: Spleen-based proteogenomics reveals that Escherichia coli infection induces activation of phagosome maturation pathway in chicken
Source: Virulence. 2023 Jan 4;14(1):2150453. doi: 10.1080/21505594.2022.2150453 (PMC9817119; doi:10.1080/21505594.2022.2150453)
Supplement: Supplemental Material [file KVIR_A_2150453_SM7109.zip › supplementary/Supplement figures-Proof.pptx]

## Slide 1
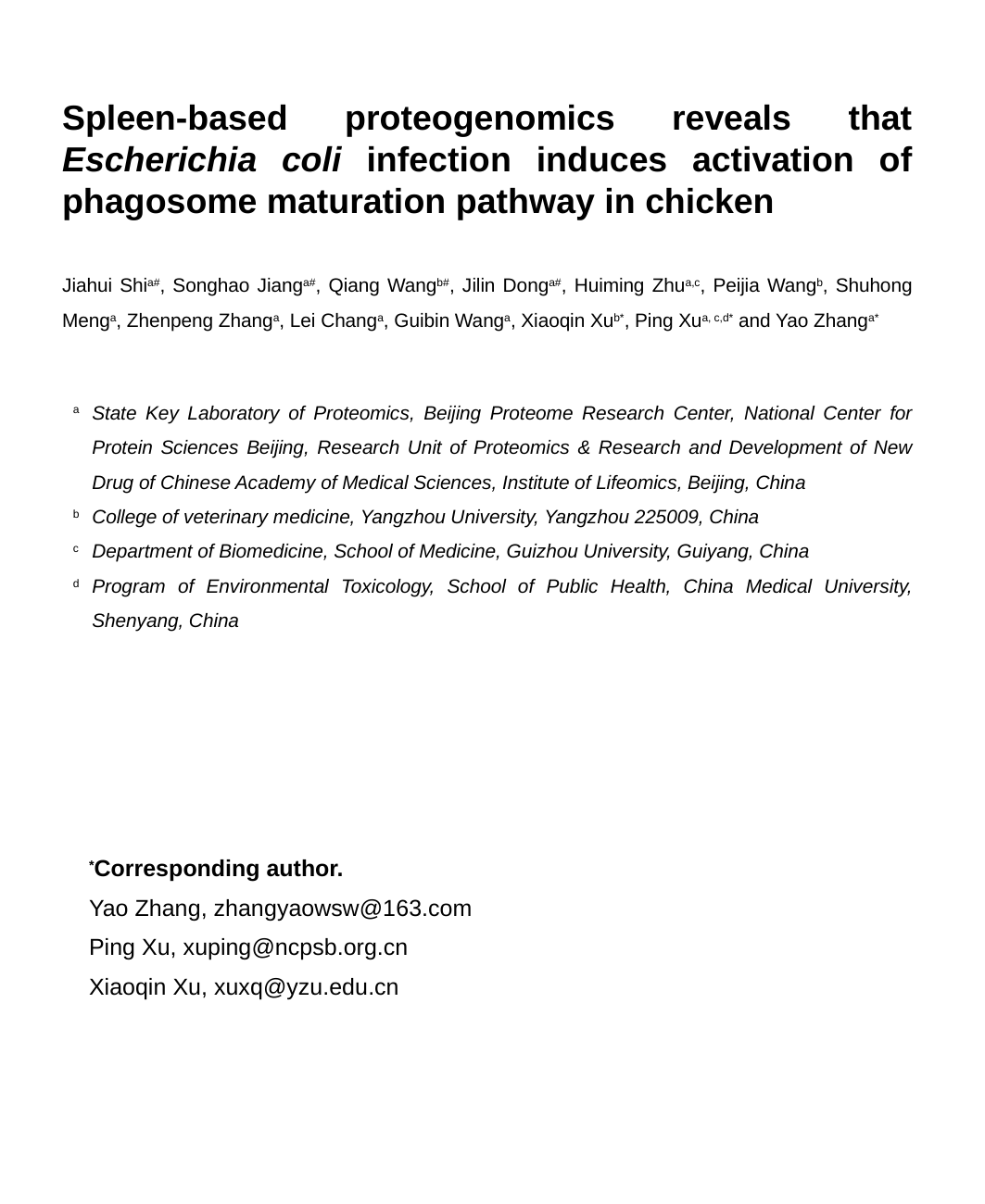

Spleen-based proteogenomics reveals that Escherichia coli infection induces activation of phagosome maturation pathway in chicken
Jiahui Shia#, Songhao Jianga#, Qiang Wangb#, Jilin Donga#, Huiming Zhua,c, Peijia Wangb, Shuhong Menga, Zhenpeng Zhanga, Lei Changa, Guibin Wanga, Xiaoqin Xub*, Ping Xua, c,d* and Yao Zhanga*
a	State Key Laboratory of Proteomics, Beijing Proteome Research Center, National Center for Protein Sciences Beijing, Research Unit of Proteomics & Research and Development of New Drug of Chinese Academy of Medical Sciences, Institute of Lifeomics, Beijing, China
b	College of veterinary medicine, Yangzhou University, Yangzhou 225009, China
c	Department of Biomedicine, School of Medicine, Guizhou University, Guiyang, China
d	Program of Environmental Toxicology, School of Public Health, China Medical University, Shenyang, China
*Corresponding author.
Yao Zhang, zhangyaowsw@163.com
Ping Xu, xuping@ncpsb.org.cn
Xiaoqin Xu, xuxq@yzu.edu.cn

## Slide 2
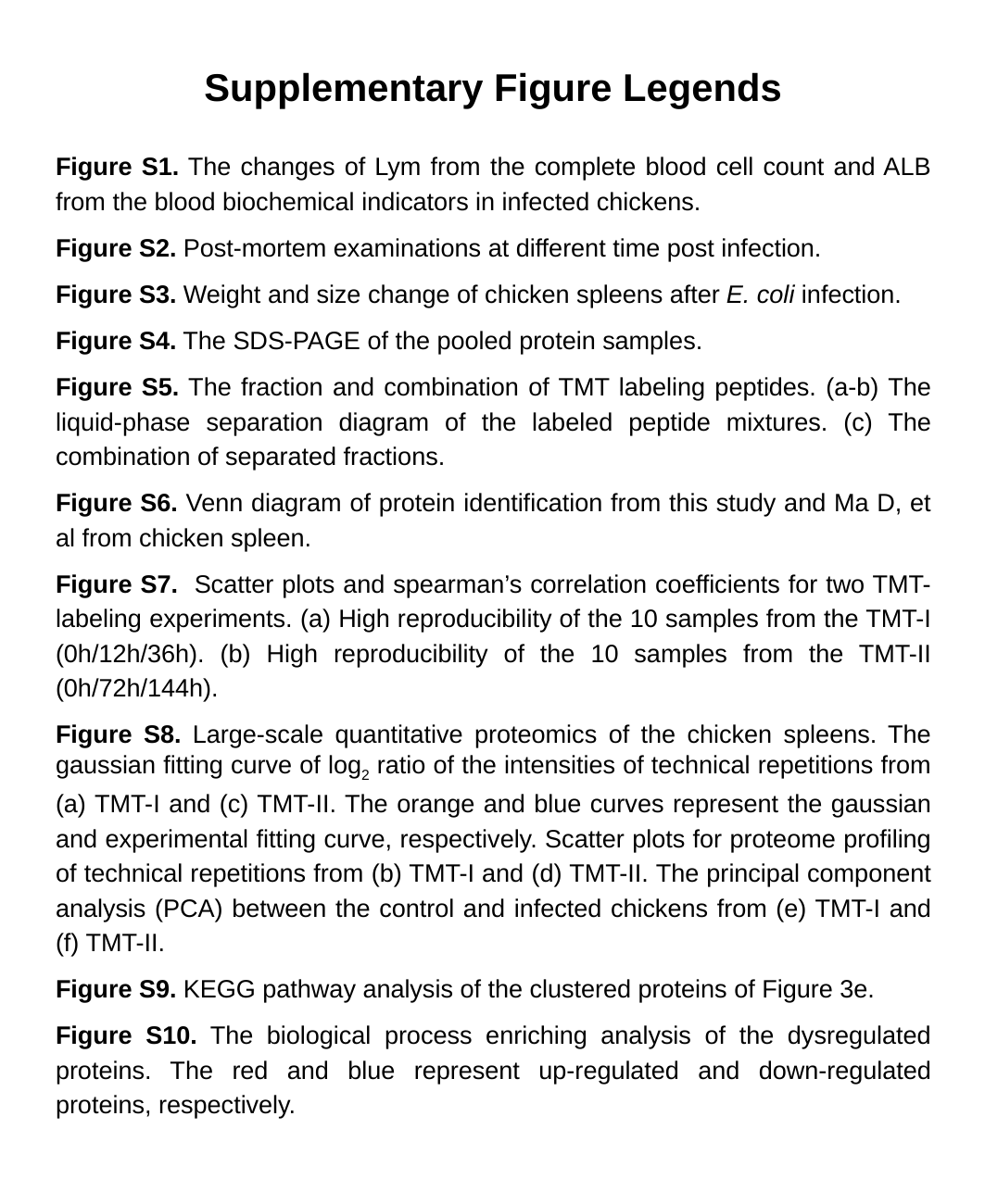

Supplementary Figure Legends
Figure S1. The changes of Lym from the complete blood cell count and ALB from the blood biochemical indicators in infected chickens.
Figure S2. Post-mortem examinations at different time post infection.
Figure S3. Weight and size change of chicken spleens after E. coli infection.
Figure S4. The SDS-PAGE of the pooled protein samples.
Figure S5. The fraction and combination of TMT labeling peptides. (a-b) The liquid-phase separation diagram of the labeled peptide mixtures. (c) The combination of separated fractions.
Figure S6. Venn diagram of protein identification from this study and Ma D, et al from chicken spleen.
Figure S7. Scatter plots and spearman’s correlation coefficients for two TMT-labeling experiments. (a) High reproducibility of the 10 samples from the TMT-I (0h/12h/36h). (b) High reproducibility of the 10 samples from the TMT-II (0h/72h/144h).
Figure S8. Large-scale quantitative proteomics of the chicken spleens. The gaussian fitting curve of log2 ratio of the intensities of technical repetitions from (a) TMT-I and (c) TMT-II. The orange and blue curves represent the gaussian and experimental fitting curve, respectively. Scatter plots for proteome profiling of technical repetitions from (b) TMT-I and (d) TMT-II. The principal component analysis (PCA) between the control and infected chickens from (e) TMT-I and (f) TMT-II.
Figure S9. KEGG pathway analysis of the clustered proteins of Figure 3e.
Figure S10. The biological process enriching analysis of the dysregulated proteins. The red and blue represent up-regulated and down-regulated proteins, respectively.

## Slide 3
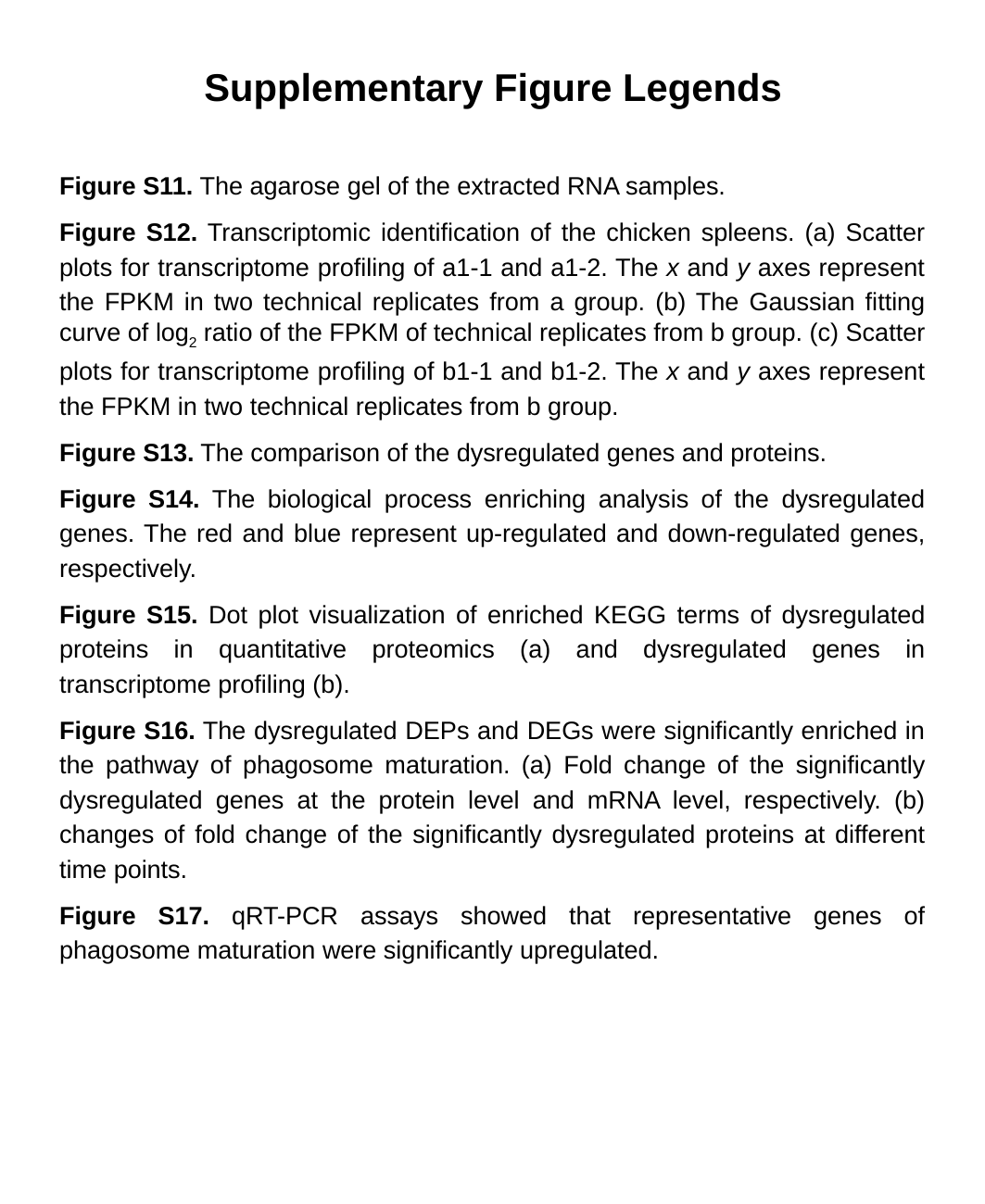

Supplementary Figure Legends
Figure S11. The agarose gel of the extracted RNA samples.
Figure S12. Transcriptomic identification of the chicken spleens. (a) Scatter plots for transcriptome profiling of a1-1 and a1-2. The x and y axes represent the FPKM in two technical replicates from a group. (b) The Gaussian fitting curve of log2 ratio of the FPKM of technical replicates from b group. (c) Scatter plots for transcriptome profiling of b1-1 and b1-2. The x and y axes represent the FPKM in two technical replicates from b group.
Figure S13. The comparison of the dysregulated genes and proteins.
Figure S14. The biological process enriching analysis of the dysregulated genes. The red and blue represent up-regulated and down-regulated genes, respectively.
Figure S15. Dot plot visualization of enriched KEGG terms of dysregulated proteins in quantitative proteomics (a) and dysregulated genes in transcriptome profiling (b).
Figure S16. The dysregulated DEPs and DEGs were significantly enriched in the pathway of phagosome maturation. (a) Fold change of the significantly dysregulated genes at the protein level and mRNA level, respectively. (b) changes of fold change of the significantly dysregulated proteins at different time points.
Figure S17. qRT-PCR assays showed that representative genes of phagosome maturation were significantly upregulated.

## Slide 4
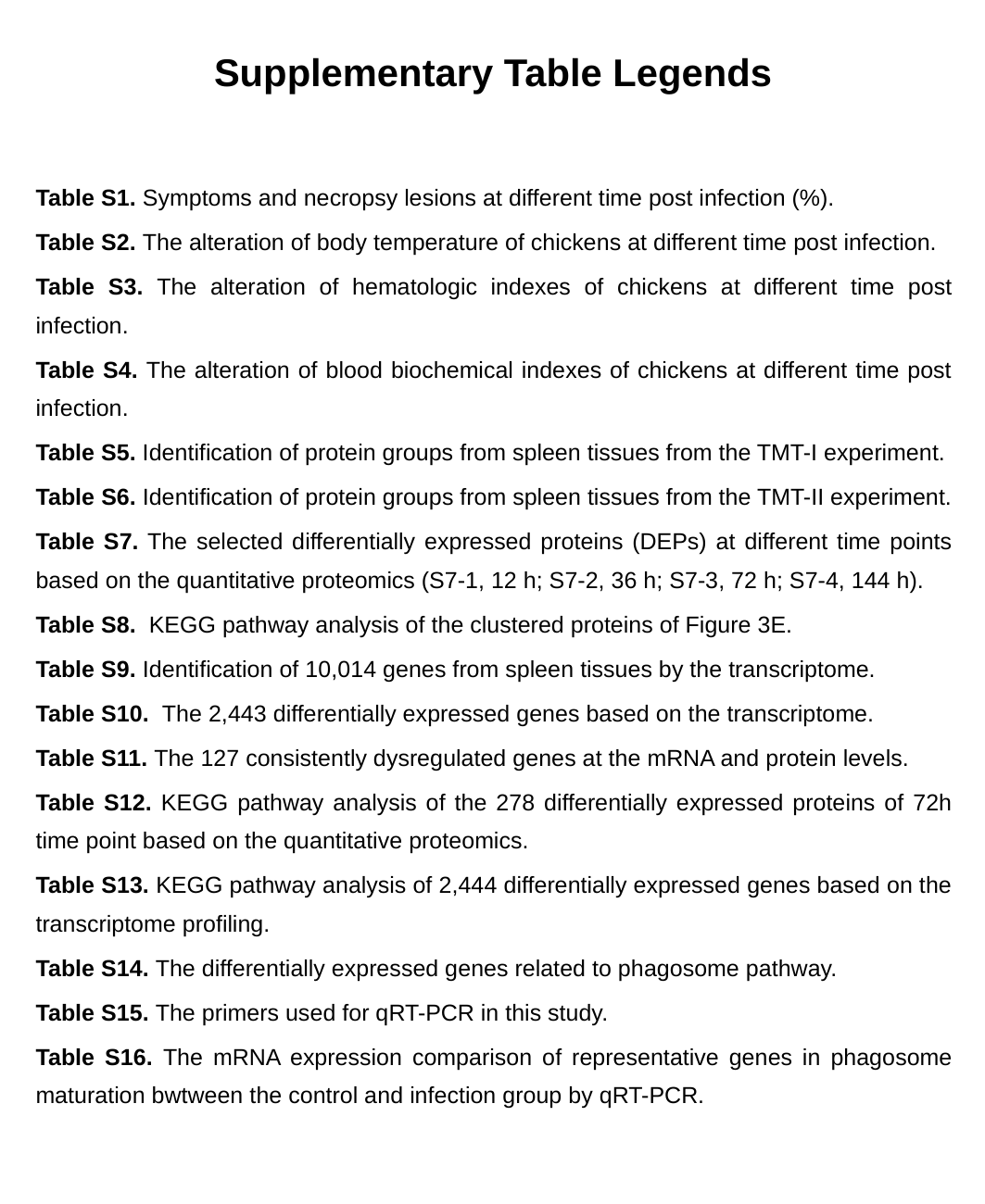

Supplementary Table Legends
Table S1. Symptoms and necropsy lesions at different time post infection (%).
Table S2. The alteration of body temperature of chickens at different time post infection.
Table S3. The alteration of hematologic indexes of chickens at different time post infection.
Table S4. The alteration of blood biochemical indexes of chickens at different time post infection.
Table S5. Identification of protein groups from spleen tissues from the TMT-I experiment.
Table S6. Identification of protein groups from spleen tissues from the TMT-II experiment.
Table S7. The selected differentially expressed proteins (DEPs) at different time points based on the quantitative proteomics (S7-1, 12 h; S7-2, 36 h; S7-3, 72 h; S7-4, 144 h).
Table S8. KEGG pathway analysis of the clustered proteins of Figure 3E.
Table S9. Identification of 10,014 genes from spleen tissues by the transcriptome.
Table S10. The 2,443 differentially expressed genes based on the transcriptome.
Table S11. The 127 consistently dysregulated genes at the mRNA and protein levels.
Table S12. KEGG pathway analysis of the 278 differentially expressed proteins of 72h time point based on the quantitative proteomics.
Table S13. KEGG pathway analysis of 2,444 differentially expressed genes based on the transcriptome profiling.
Table S14. The differentially expressed genes related to phagosome pathway.
Table S15. The primers used for qRT-PCR in this study.
Table S16. The mRNA expression comparison of representative genes in phagosome maturation bwtween the control and infection group by qRT-PCR.

## Slide 5
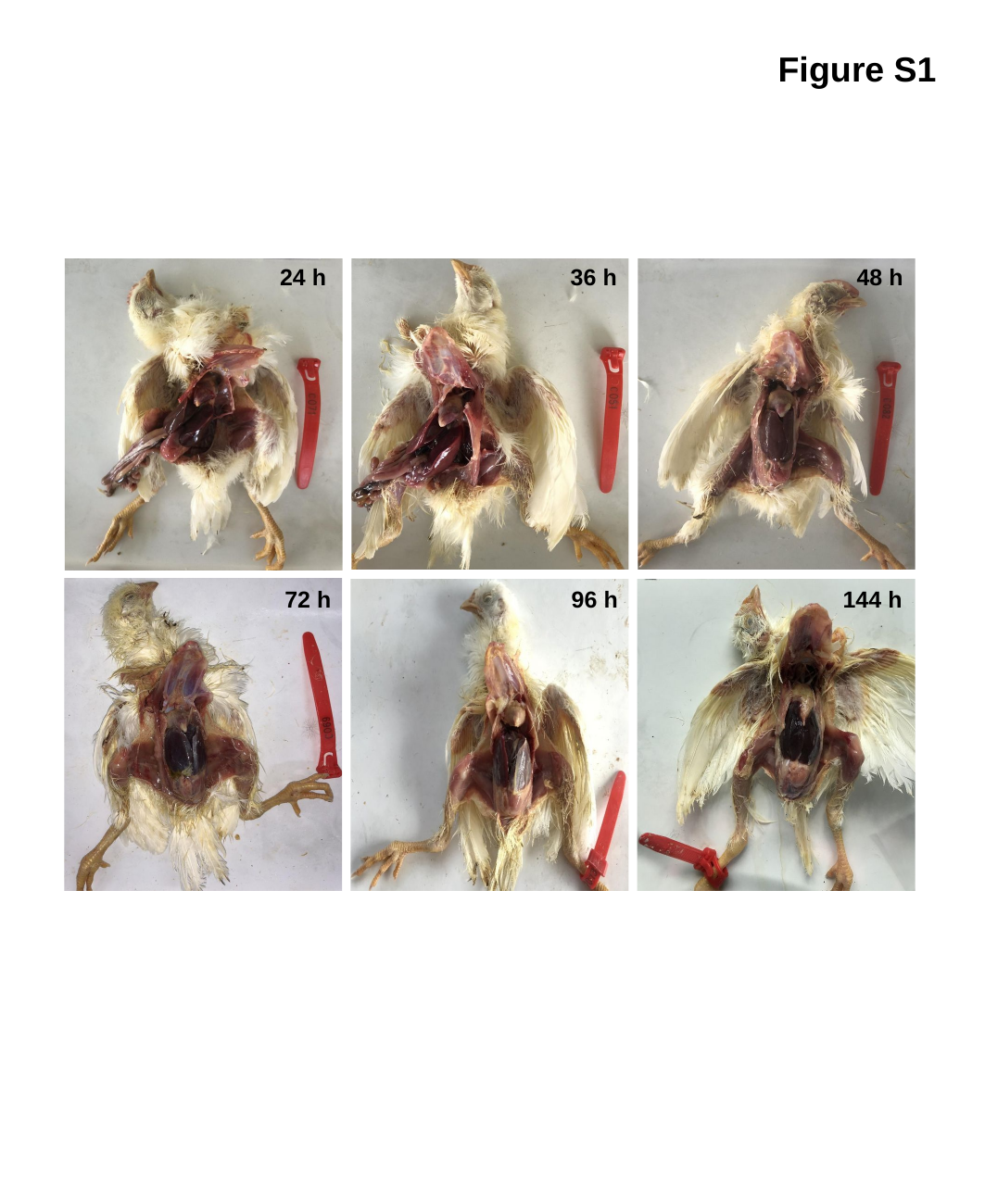

Figure S1
24 h
36 h
48 h
72 h
96 h
144 h

## Slide 6
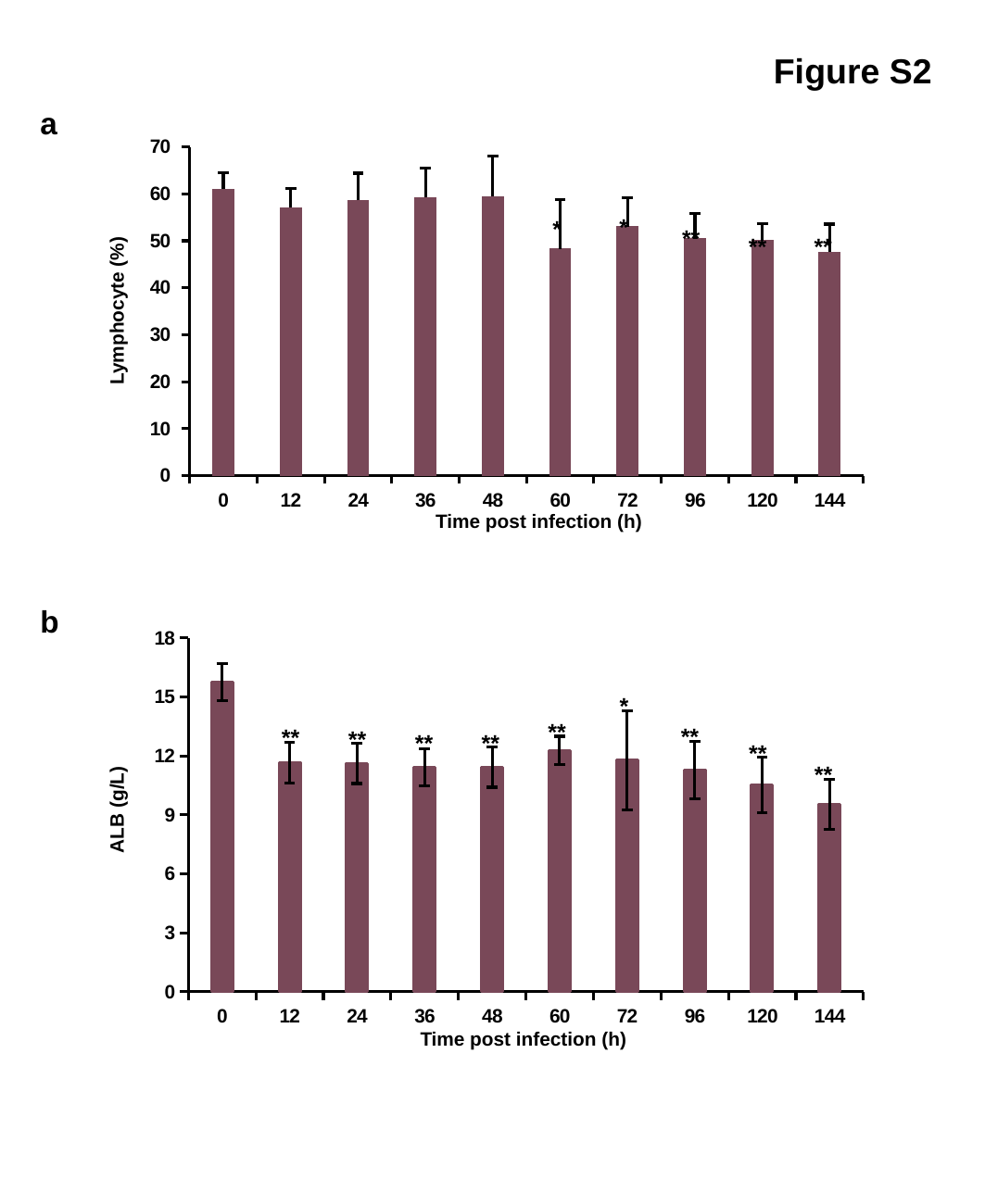

Figure S2
a
### Chart
| Category | 均值 |
|---|---|
| 0 | 61.0 |
| 12 | 57.0 |
| 24 | 58.57142857142857 |
| 36 | 59.166666666666664 |
| 48 | 59.4 |
| 60 | 48.285714285714285 |
| 72 | 53.0 |
| 96 | 50.4 |
| 120 | 50.0 |
| 144 | 47.625 |*
*
**
**
**
Lymphocyte (%)
Time post infection (h)
b
### Chart
| Category | |
|---|---|
| 0 | 15.741176470588233 |
| 12 | 11.66 |
| 24 | 11.625000000000002 |
| 36 | 11.416666666666666 |
| 48 | 11.424999999999999 |
| 60 | 12.280000000000001 |
| 72 | 11.78 |
| 96 | 11.286666666666669 |
| 120 | 10.514285714285714 |
| 144 | 9.537500000000001 |*
**
**
**
**
**
**
**
**
ALB (g/L)
Time post infection (h)

## Slide 7
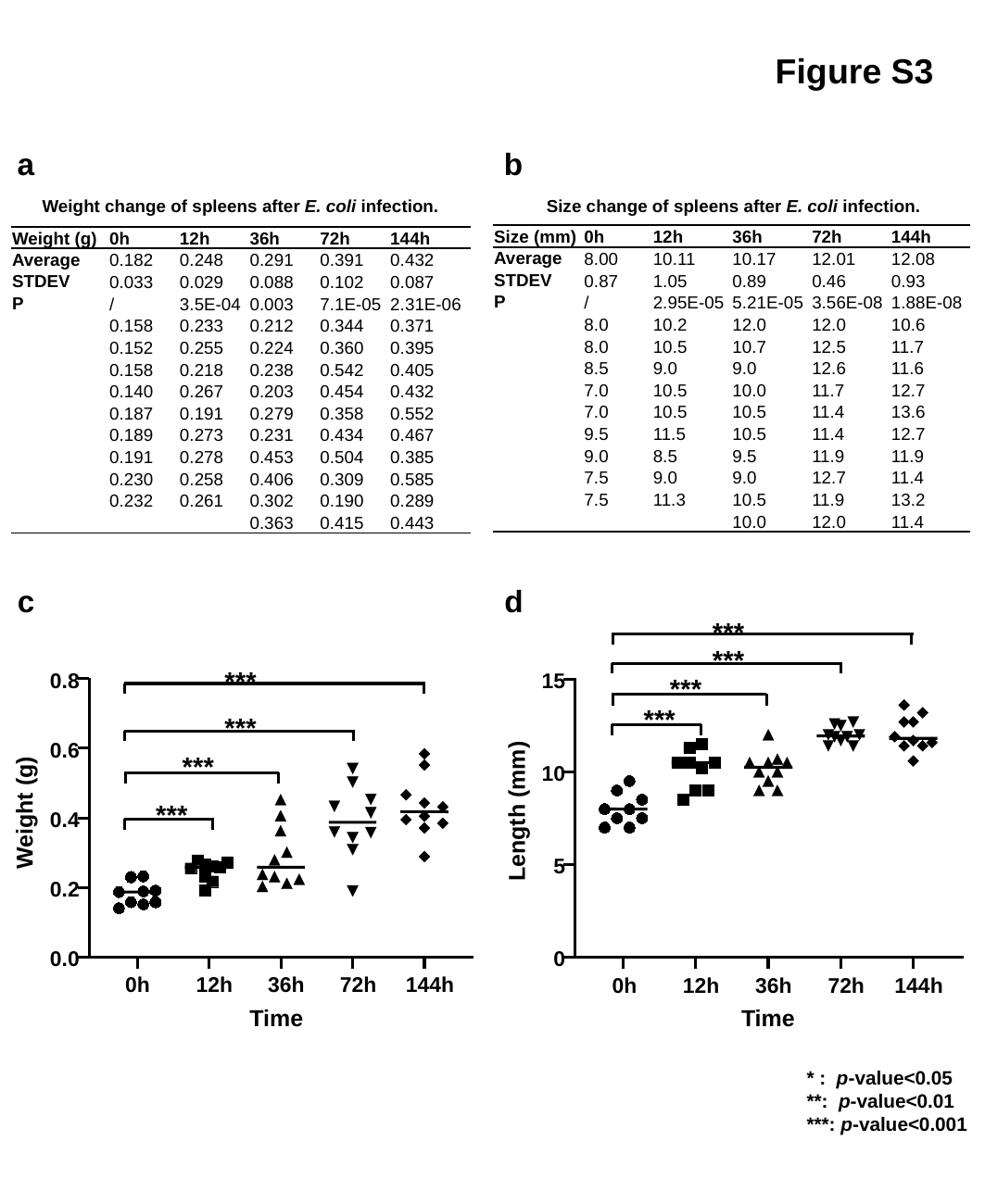

Figure S3
a
b
Size change of spleens after E. coli infection.
Weight change of spleens after E. coli infection.
| Size (mm) | 0h | 12h | 36h | 72h | 144h |
| --- | --- | --- | --- | --- | --- |
| Average | 8.00 | 10.11 | 10.17 | 12.01 | 12.08 |
| STDEV | 0.87 | 1.05 | 0.89 | 0.46 | 0.93 |
| P | / | 2.95E-05 | 5.21E-05 | 3.56E-08 | 1.88E-08 |
| | 8.0 | 10.2 | 12.0 | 12.0 | 10.6 |
| | 8.0 | 10.5 | 10.7 | 12.5 | 11.7 |
| | 8.5 | 9.0 | 9.0 | 12.6 | 11.6 |
| | 7.0 | 10.5 | 10.0 | 11.7 | 12.7 |
| | 7.0 | 10.5 | 10.5 | 11.4 | 13.6 |
| | 9.5 | 11.5 | 10.5 | 11.4 | 12.7 |
| | 9.0 | 8.5 | 9.5 | 11.9 | 11.9 |
| | 7.5 | 9.0 | 9.0 | 12.7 | 11.4 |
| | 7.5 | 11.3 | 10.5 | 11.9 | 13.2 |
| | | | 10.0 | 12.0 | 11.4 |
| Weight (g) | 0h | 12h | 36h | 72h | 144h |
| --- | --- | --- | --- | --- | --- |
| Average | 0.182 | 0.248 | 0.291 | 0.391 | 0.432 |
| STDEV | 0.033 | 0.029 | 0.088 | 0.102 | 0.087 |
| P | / | 3.5E-04 | 0.003 | 7.1E-05 | 2.31E-06 |
| | 0.158 | 0.233 | 0.212 | 0.344 | 0.371 |
| | 0.152 | 0.255 | 0.224 | 0.360 | 0.395 |
| | 0.158 | 0.218 | 0.238 | 0.542 | 0.405 |
| | 0.140 | 0.267 | 0.203 | 0.454 | 0.432 |
| | 0.187 | 0.191 | 0.279 | 0.358 | 0.552 |
| | 0.189 | 0.273 | 0.231 | 0.434 | 0.467 |
| | 0.191 | 0.278 | 0.453 | 0.504 | 0.385 |
| | 0.230 | 0.258 | 0.406 | 0.309 | 0.585 |
| | 0.232 | 0.261 | 0.302 | 0.190 | 0.289 |
| | | | 0.363 | 0.415 | 0.443 |
c
d
***
***
***
15
***
10
Length (mm)
5
0
0h
12h
36h
72h
144h
Time
***
0.8
***
0.6
***
***
Weight (g)
0.4
0.2
0.0
0h
12h
36h
72h
144h
Time
* : p-value<0.05
**: p-value<0.01
***: p-value<0.001

## Slide 8
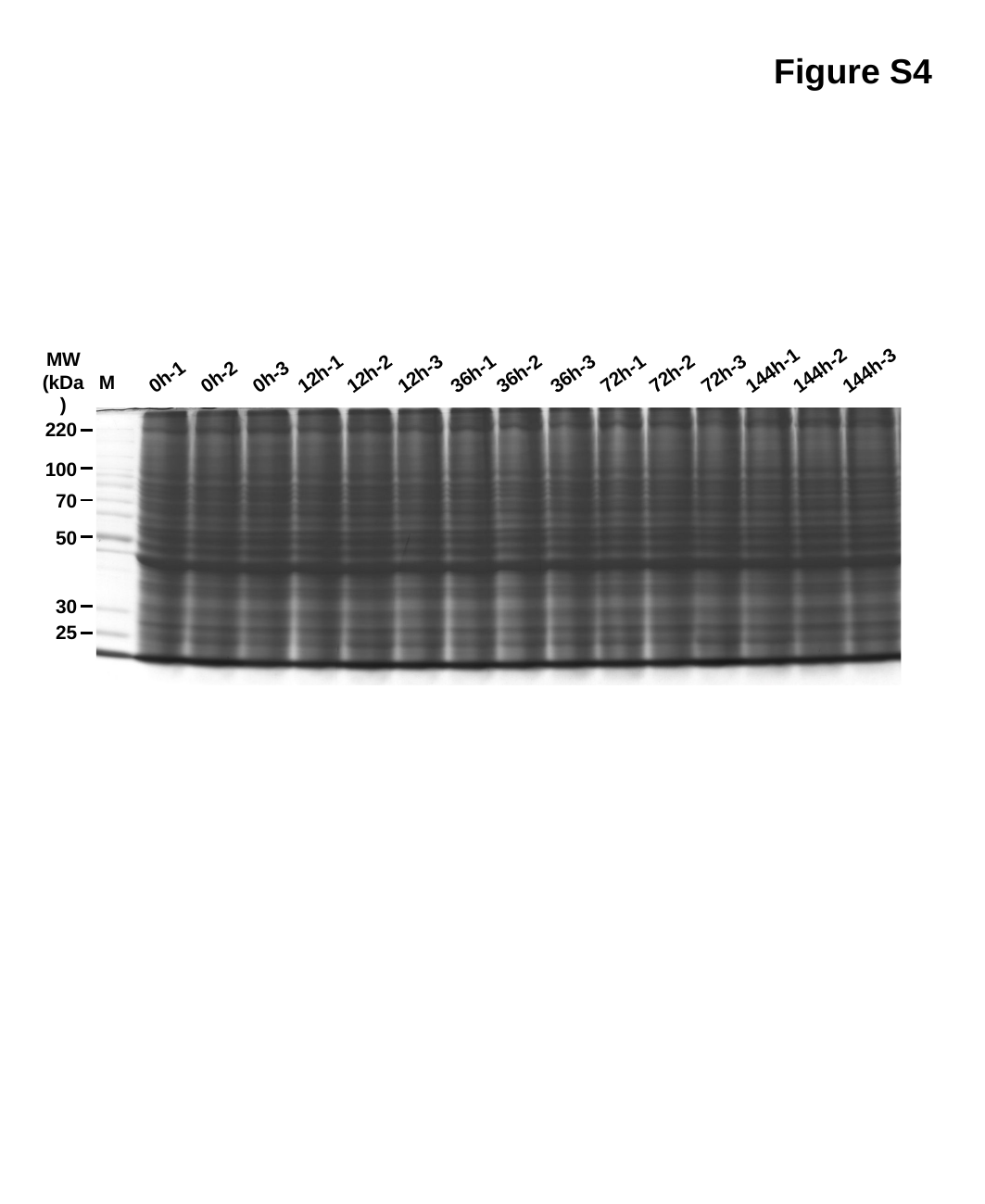

Figure S4
MW
(kDa)
144h-3
144h-1
144h-2
12h-1
12h-2
12h-3
36h-1
36h-2
36h-3
72h-1
72h-2
72h-3
0h-1
0h-2
0h-3
M
220
100
70
50
30
25

## Slide 9
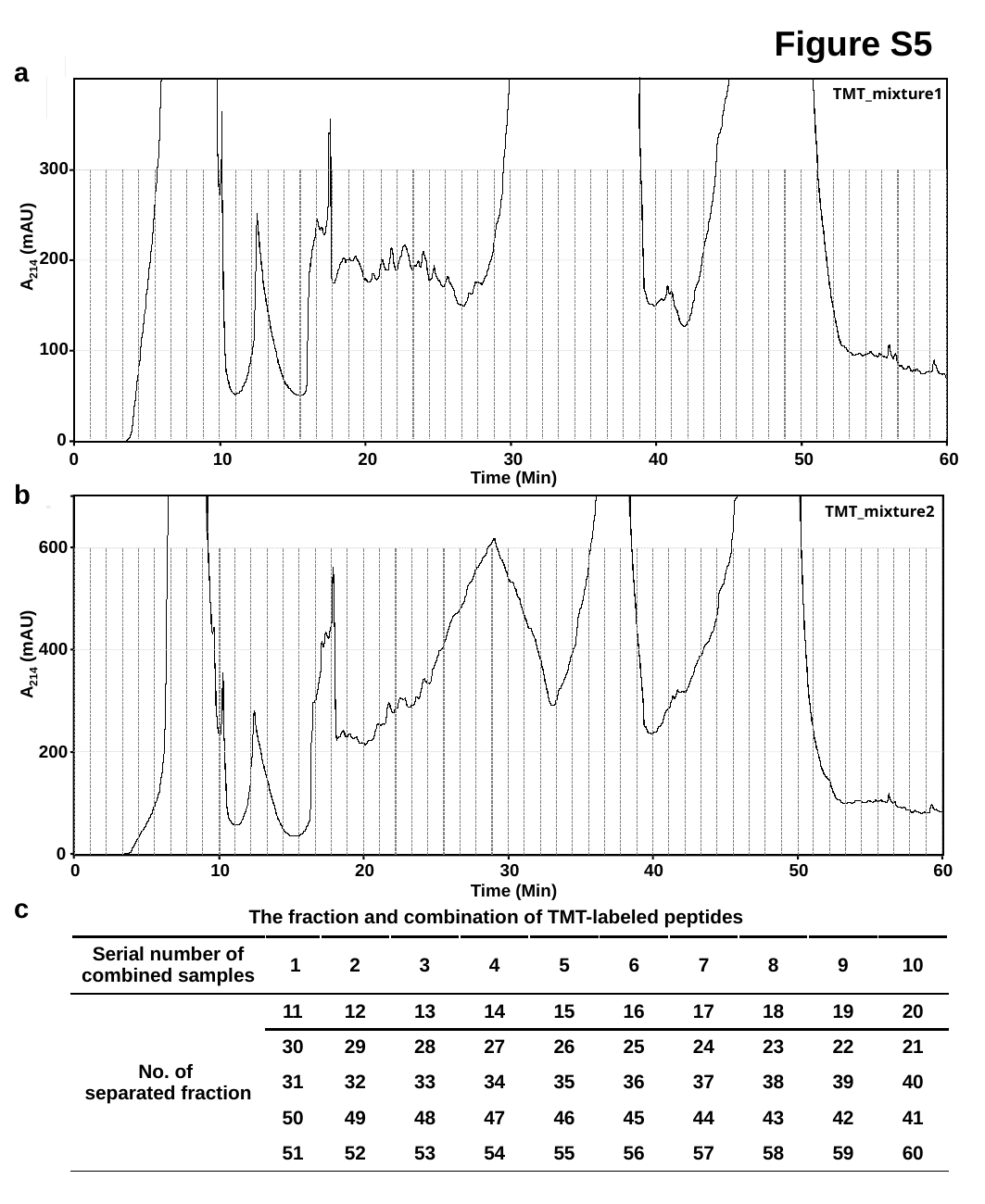

Figure S5
a
TMT_mixture1
300
A214 (mAU)
200
100
0
0
10
20
30
40
50
60
Time (Min)
b
TMT_mixture2
600
400
A214 (mAU)
200
0
0
10
20
30
40
50
60
Time (Min)
c
The fraction and combination of TMT-labeled peptides
| Serial number of combined samples | 1 | 2 | 3 | 4 | 5 | 6 | 7 | 8 | 9 | 10 |
| --- | --- | --- | --- | --- | --- | --- | --- | --- | --- | --- |
| No. of separated fraction | 11 | 12 | 13 | 14 | 15 | 16 | 17 | 18 | 19 | 20 |
| | 30 | 29 | 28 | 27 | 26 | 25 | 24 | 23 | 22 | 21 |
| | 31 | 32 | 33 | 34 | 35 | 36 | 37 | 38 | 39 | 40 |
| | 50 | 49 | 48 | 47 | 46 | 45 | 44 | 43 | 42 | 41 |
| | 51 | 52 | 53 | 54 | 55 | 56 | 57 | 58 | 59 | 60 |

## Slide 10
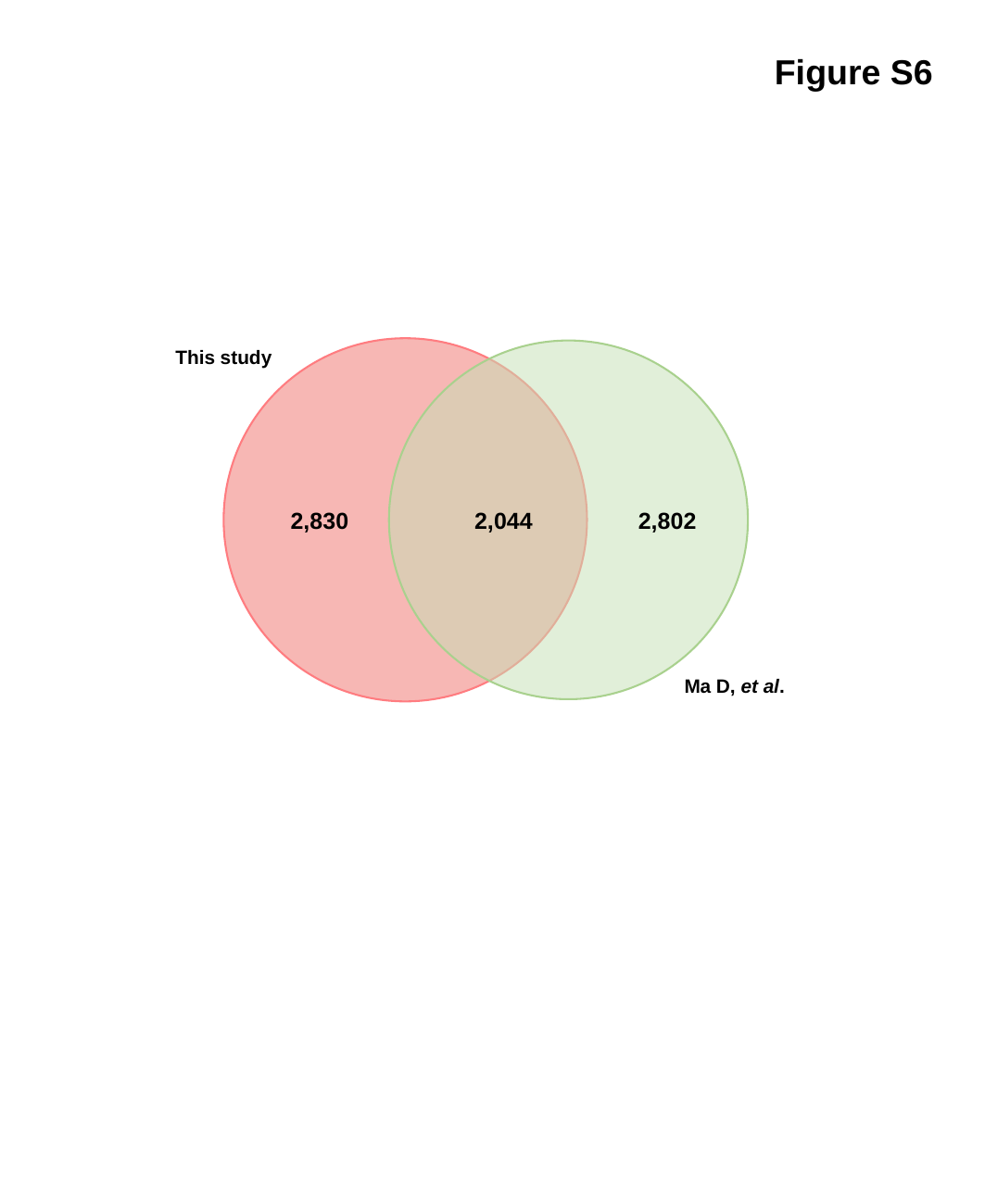

Figure S6
This study
2,830
2,044
2,802
Ma D, et al.

## Slide 11
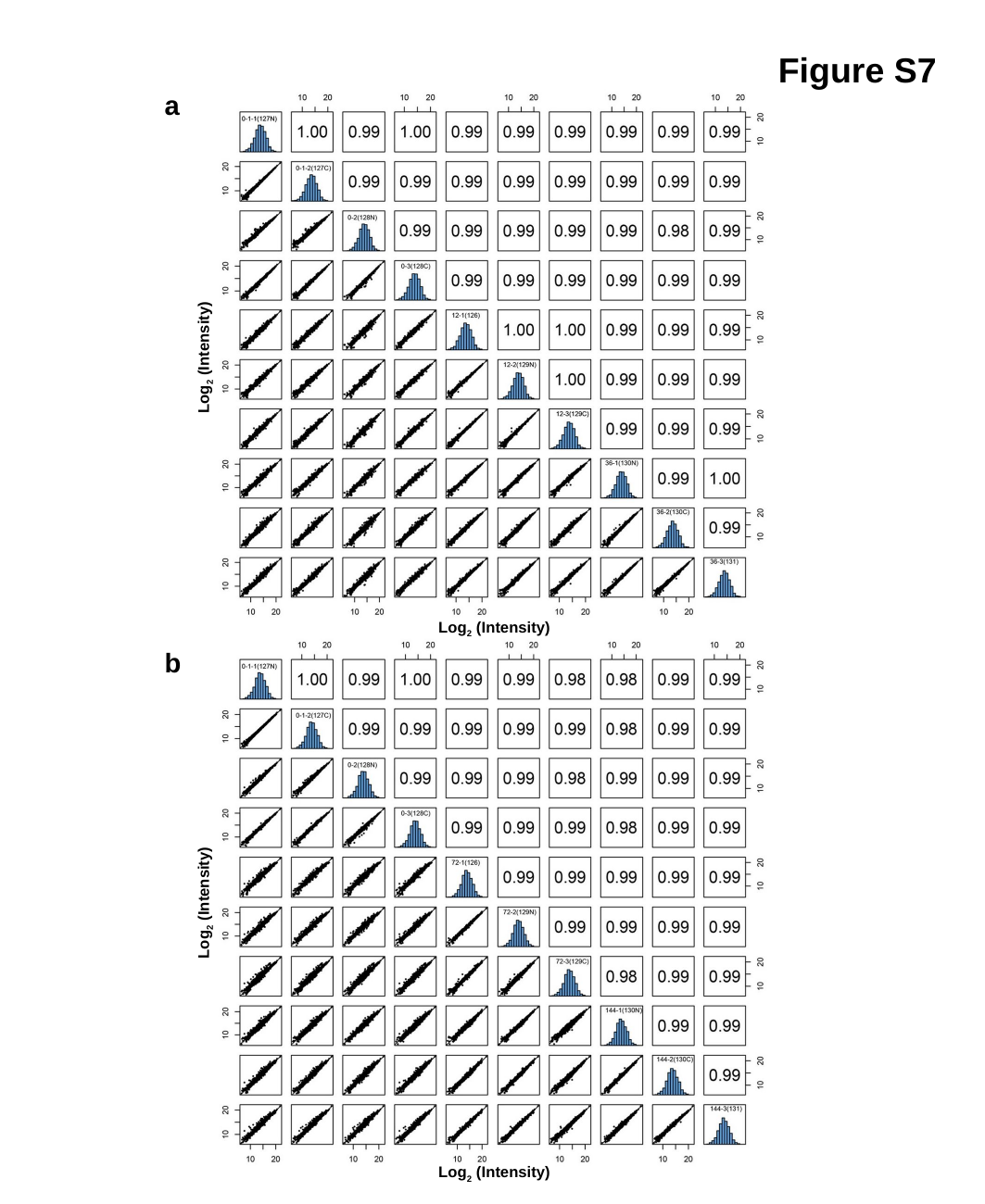

Figure S7
a
Log2 (Intensity)
Log2 (Intensity)
b
Log2 (Intensity)
Log2 (Intensity)

## Slide 12
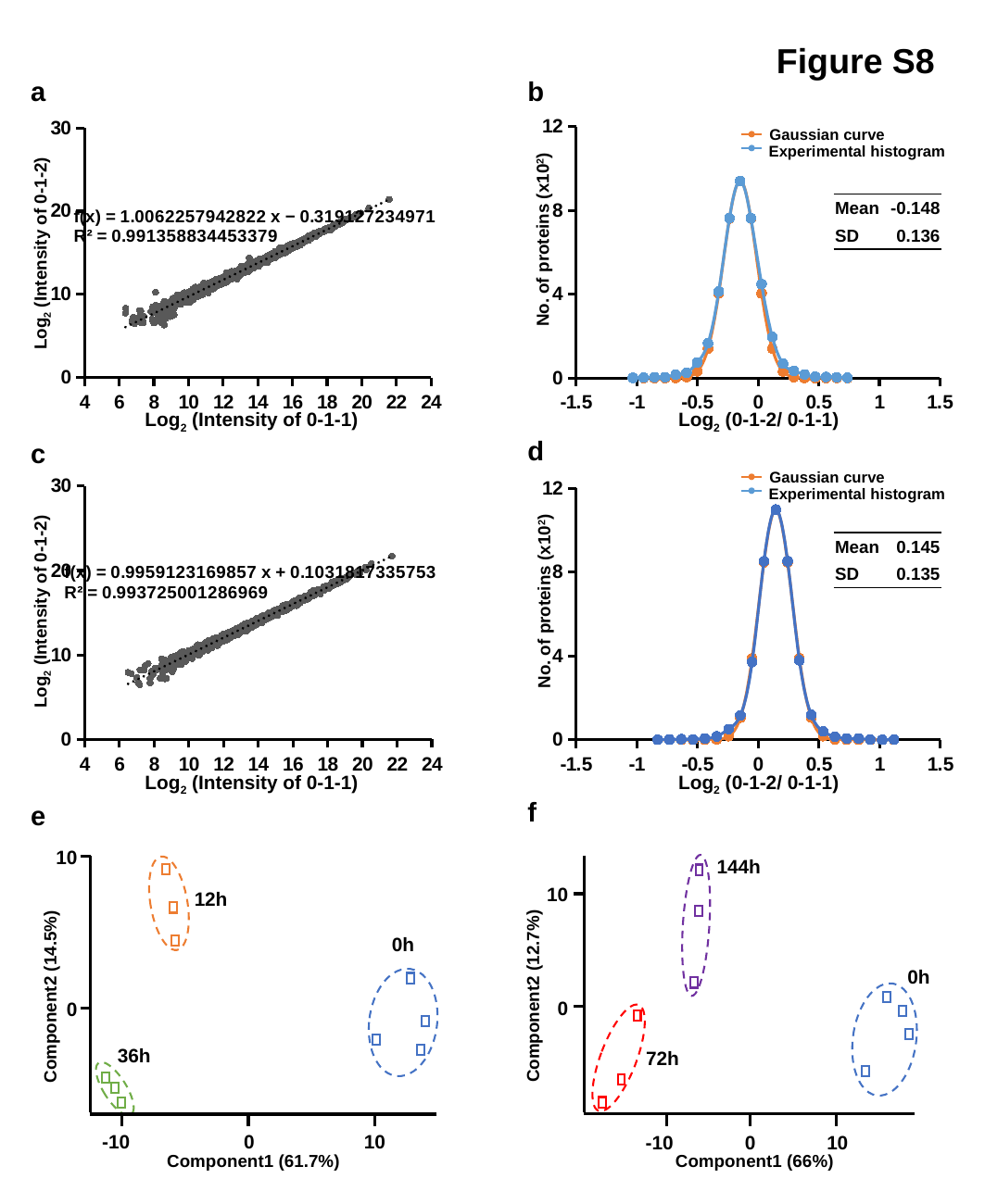

Figure S8
a
b
### Chart
| Category | | |
|---|---|---|
### Chart
| Category | 0-1-2(127C) |
|---|---|Gaussian curve
Experimental histogram
| Mean | -0.148 |
| --- | --- |
| SD | 0.136 |
No. of proteins (x102)
Log2 (Intensity of 0-1-2)
Log2 (Intensity of 0-1-1)
Log2 (0-1-2/ 0-1-1)
d
c
Gaussian curve
Experimental histogram
### Chart
| Category | 0-1-2(127C) |
|---|---|
### Chart
| Category | | |
|---|---|---|| Mean | 0.145 |
| --- | --- |
| SD | 0.135 |
No. of proteins (x102)
Log2 (Intensity of 0-1-2)
Log2 (Intensity of 0-1-1)
Log2 (0-1-2/ 0-1-1)
f
e
10
144h
0h
72h
10
Component2 (12.7%)
0
-10
0
10
Component1 (66%)
12h
0h
36h
Component2 (14.5%)
0
-10
0
10
Component1 (61.7%)

## Slide 13
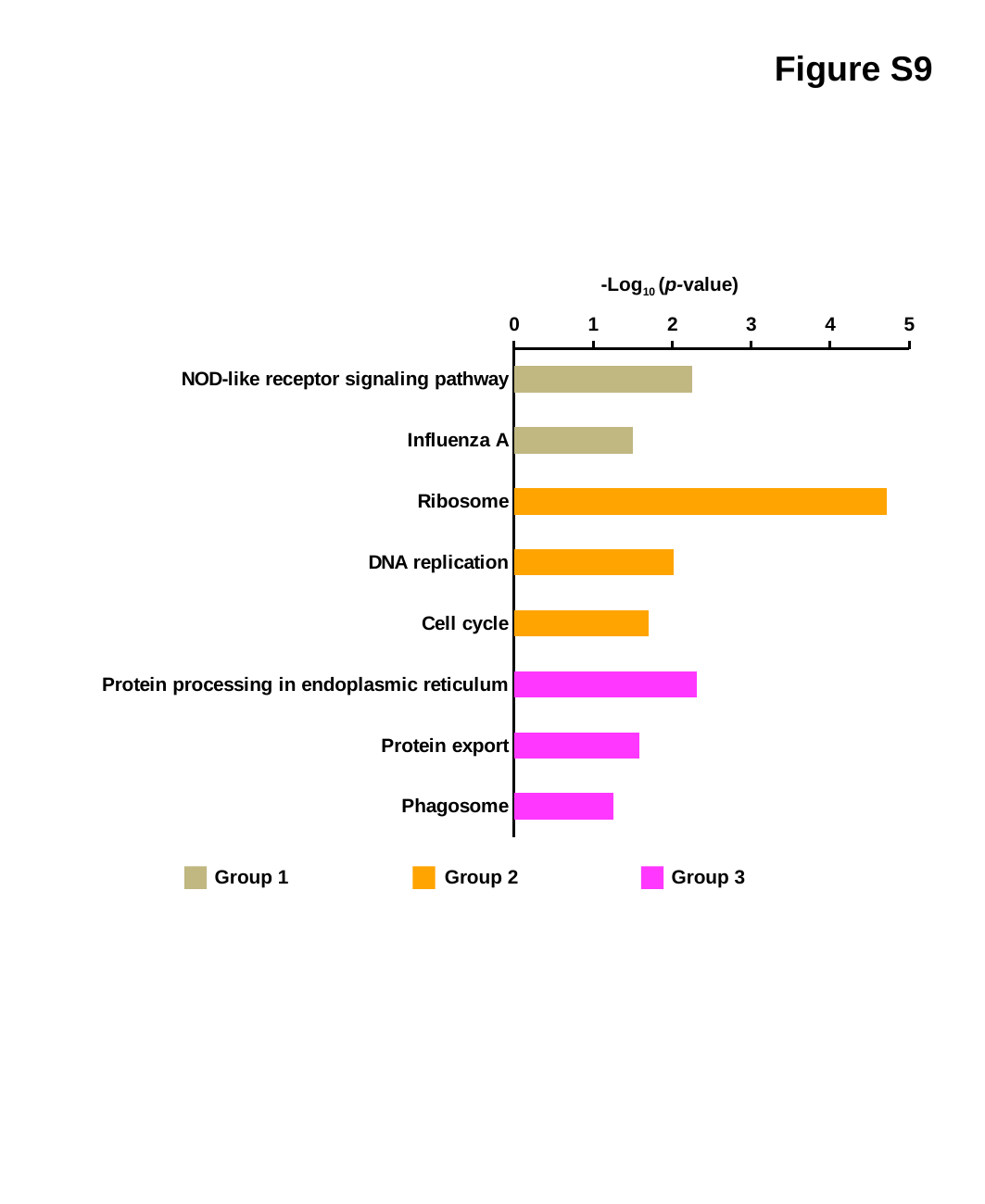

Figure S9
-Log10 (p-value)
### Chart
| Category | |
|---|---|
| NOD-like receptor signaling pathway | 2.2518596997622495 |
| Influenza A | 1.5050477274387621 |
| Ribosome | 4.713240209176932 |
| DNA replication | 2.020283002838685 |
| Cell cycle | 1.7071700572670208 |
| Protein processing in endoplasmic reticulum | 2.3155911441332027 |
| Protein export | 1.5831574487084845 |
| Phagosome | 1.2528818180259595 |Group 1
Group 2
Group 3

## Slide 14
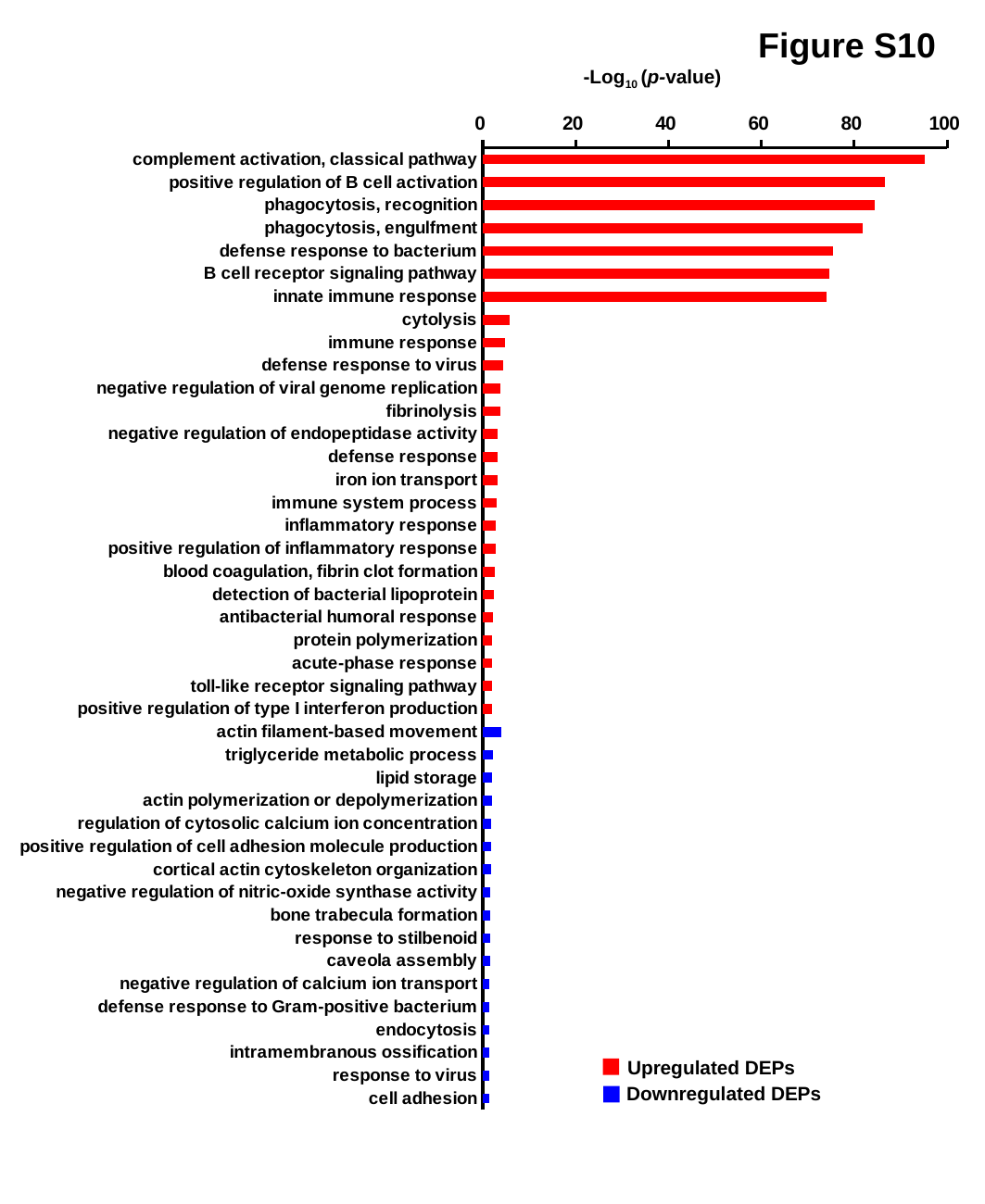

Figure S10
-Log10 (p-value)
### Chart
| Category | |
|---|---|
| complement activation, classical pathway | 95.12500592394241 |
| positive regulation of B cell activation | 86.68449664724305 |
| phagocytosis, recognition | 84.41572147178977 |
| phagocytosis, engulfment | 81.88642701648025 |
| defense response to bacterium | 75.4222173668136 |
| B cell receptor signaling pathway | 74.64481971312865 |
| innate immune response | 74.06812756048788 |
| cytolysis | 5.709452598387213 |
| immune response | 4.862519801653748 |
| defense response to virus | 4.413685709865736 |
| negative regulation of viral genome replication | 3.8589799333085217 |
| fibrinolysis | 3.84626488444312 |
| negative regulation of endopeptidase activity | 3.2917632588525167 |
| defense response | 3.248054749980201 |
| iron ion transport | 3.1938372461622984 |
| immune system process | 3.074135390226173 |
| inflammatory response | 2.741438631985762 |
| positive regulation of inflammatory response | 2.712254307330085 |
| blood coagulation, fibrin clot formation | 2.5916286306529064 |
| detection of bacterial lipoprotein | 2.4202210348929336 |
| antibacterial humoral response | 2.158502930458346 |
| protein polymerization | 2.0540234350659716 |
| acute-phase response | 2.0540234350659716 |
| toll-like receptor signaling pathway | 2.028486216796943 |
| positive regulation of type I interferon production | 1.9617721359583387 |
| actin filament-based movement | 3.9531763112594285 |
| triglyceride metabolic process | 2.203446030709469 |
| lipid storage | 1.9709960714750516 |
| actin polymerization or depolymerization | 1.9317173217045032 |
| regulation of cytosolic calcium ion concentration | 1.8586443950494527 |
| positive regulation of cell adhesion molecule production | 1.8248313471023478 |
| cortical actin cytoskeleton organization | 1.8245424593153161 |
| negative regulation of nitric-oxide synthase activity | 1.6503561858231297 |
| bone trabecula formation | 1.5270316105758721 |
| response to stilbenoid | 1.5270316105758721 |
| caveola assembly | 1.5270316105758721 |
| negative regulation of calcium ion transport | 1.4317338220578735 |
| defense response to Gram-positive bacterium | 1.4214444807528945 |
| endocytosis | 1.3723751824870662 |
| intramembranous ossification | 1.3541628632731073 |
| response to virus | 1.3139477305963807 |
| cell adhesion | 1.3125615181689132 |Upregulated DEPs
Downregulated DEPs

## Slide 15
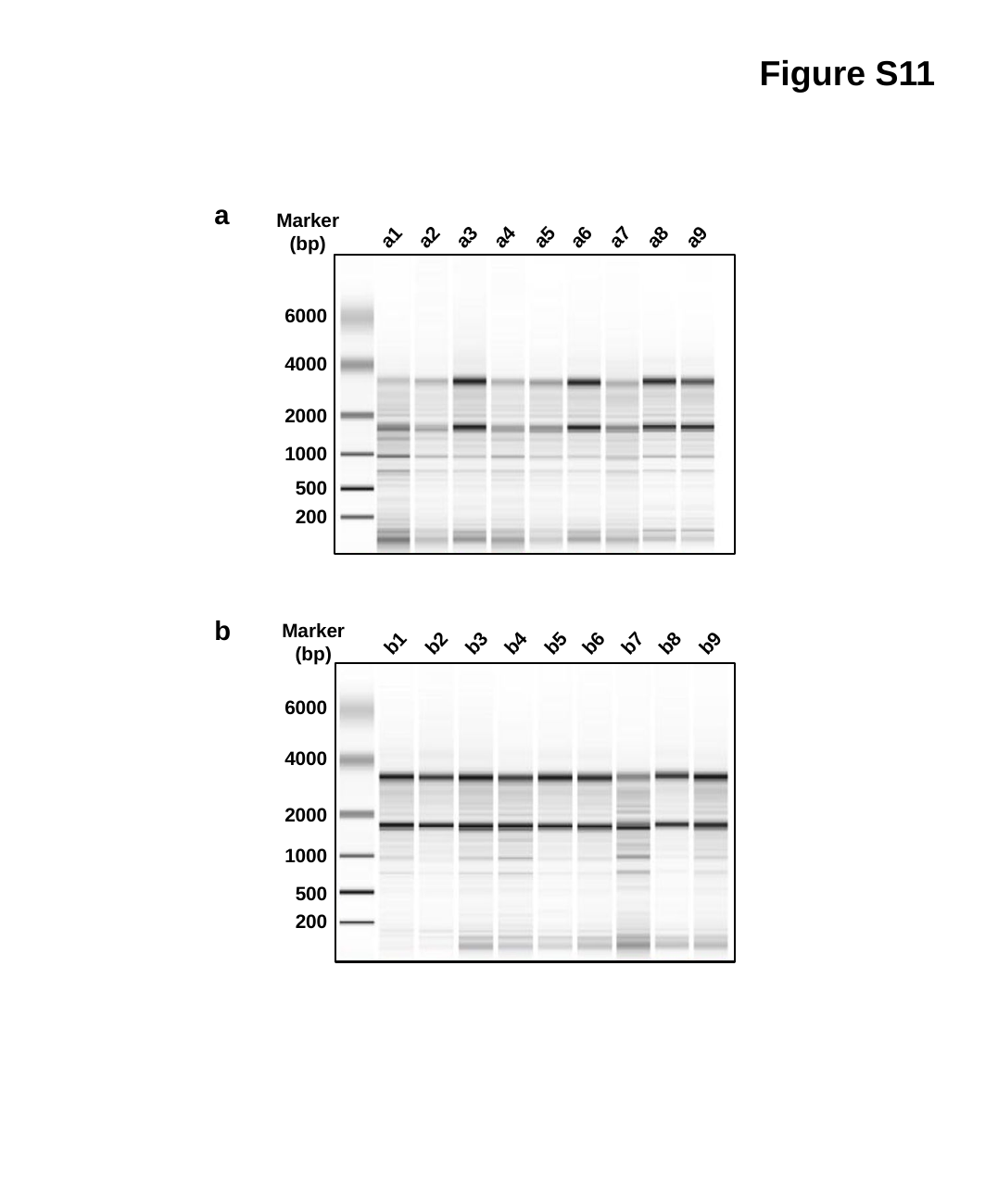

Figure S11
a
Marker
(bp)
a1
a2
a3
a4
a5
a6
a7
a8
a9
6000
4000
2000
1000
500
200
b
Marker
(bp)
b1
b2
b3
b4
b5
b6
b7
b8
b9
6000
4000
2000
1000
500
200

## Slide 16
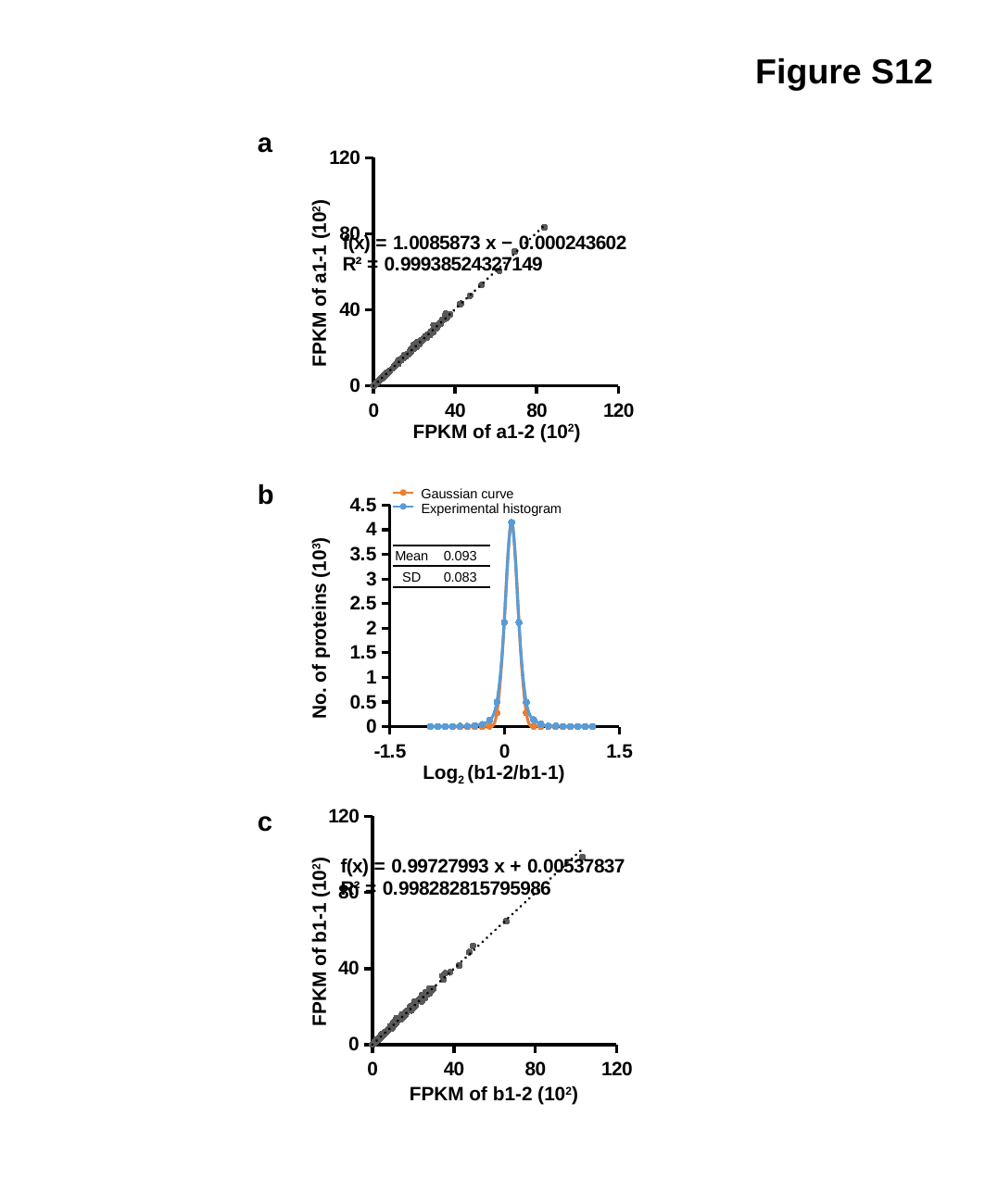

Figure S12
a
### Chart
| Category | A89_2_fpkm |
|---|---|FPKM of a1-1 (102)
FPKM of a1-2 (102)
b
Gaussian curve
### Chart
| Category | | |
|---|---|---|
Experimental histogram
| Mean | 0.093 |
| --- | --- |
| SD | 0.083 |
No. of proteins (103)
Log2 (b1-2/b1-1)
c
### Chart
| Category | B84_2_fpkm |
|---|---|FPKM of b1-1 (102)
FPKM of b1-2 (102)

## Slide 17
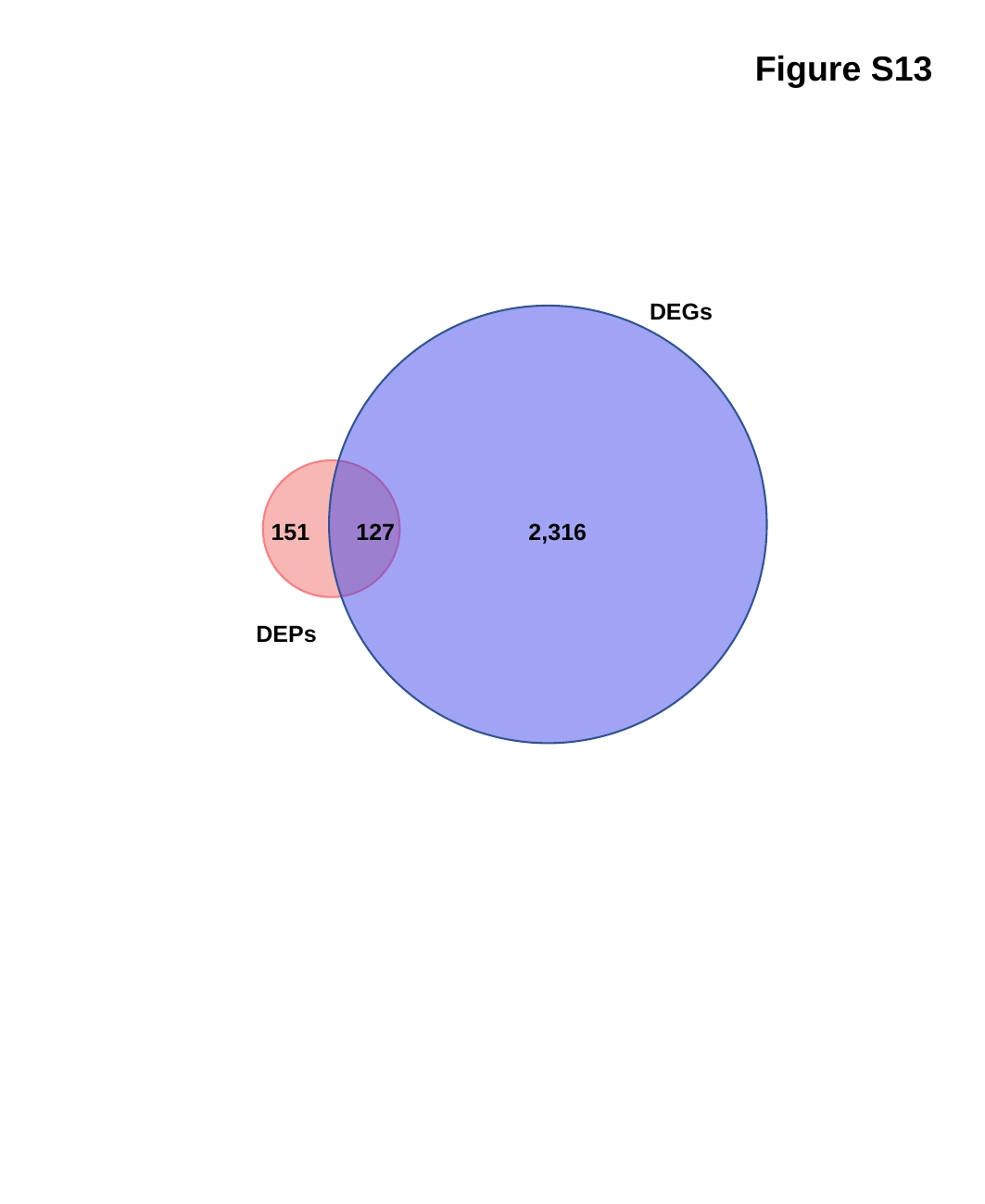

Figure S13
DEGs
151
127
2,316
DEPs

## Slide 18
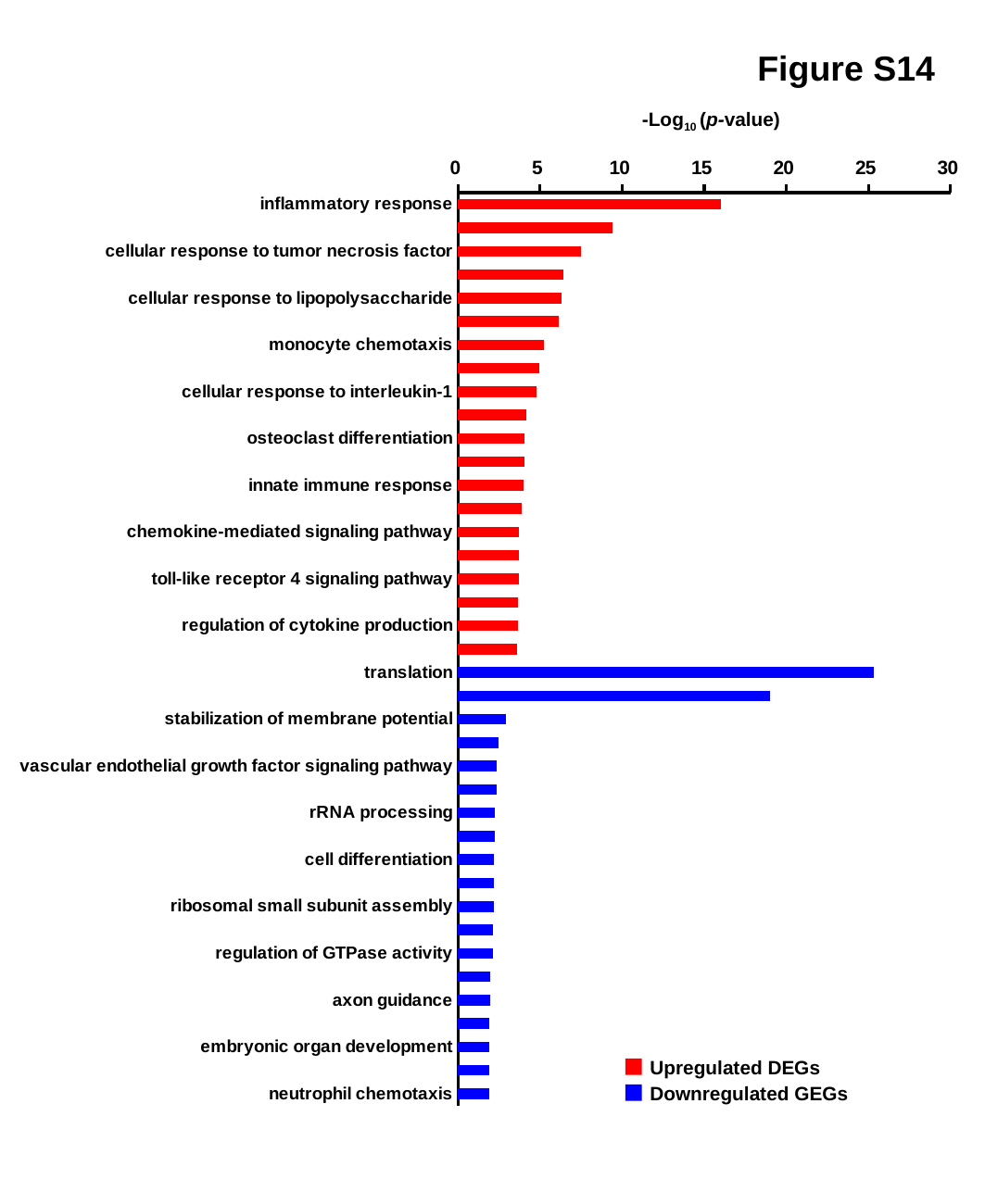

Figure S14
-Log10 (p-value)
### Chart
| Category | |
|---|---|
| inflammatory response | 16.00959614260695 |
| cytokine-mediated signaling pathway | 9.402673110016876 |
| cellular response to tumor necrosis factor | 7.493838837562203 |
| defense response to virus | 6.394925636722579 |
| cellular response to lipopolysaccharide | 6.335989177837786 |
| immune response | 6.123146906725154 |
| monocyte chemotaxis | 5.2195234241434605 |
| neutrophil chemotaxis | 4.9347656921752785 |
| cellular response to interleukin-1 | 4.797519680591218 |
| positive regulation of p38MAPK cascade | 4.186282450517605 |
| osteoclast differentiation | 4.061113638027284 |
| positive regulation of interferon-gamma production | 4.025448118972261 |
| innate immune response | 3.9903187690159045 |
| positive regulation of inflammatory response | 3.887452600585447 |
| chemokine-mediated signaling pathway | 3.716363977298959 |
| positive regulation of T cell proliferation | 3.716363977298959 |
| toll-like receptor 4 signaling pathway | 3.690616967721098 |
| cellular response to interferon-gamma | 3.667021801628167 |
| regulation of cytokine production | 3.629941407451444 |
| lymphocyte chemotaxis | 3.583044255433721 |
| translation | 25.339333915297292 |
| cytoplasmic translation | 19.034502678586712 |
| stabilization of membrane potential | 2.9235139121613085 |
| calcium-mediated signaling | 2.4585913135959006 |
| vascular endothelial growth factor signaling pathway | 2.370462252343179 |
| signal transduction by p53 class mediator | 2.370462252343179 |
| rRNA processing | 2.258553827486227 |
| positive regulation of cell proliferation | 2.2423436412471163 |
| cell differentiation | 2.174268678549701 |
| antimicrobial humoral immune response mediated by antimicrobial peptide | 2.170996402916334 |
| ribosomal small subunit assembly | 2.1573546973511473 |
| peptidyl-tyrosine phosphorylation | 2.1421682154300785 |
| regulation of GTPase activity | 2.117700825167504 |
| platelet-derived growth factor receptor signaling pathway | 1.9860805523305622 |
| axon guidance | 1.968905800233306 |
| endochondral ossification | 1.9021398646513614 |
| embryonic organ development | 1.9021398646513614 |
| multicellular organism development | 1.9021398646513614 |
| neutrophil chemotaxis | 1.8897175793083556 |Upregulated DEGs
Downregulated GEGs

## Slide 19
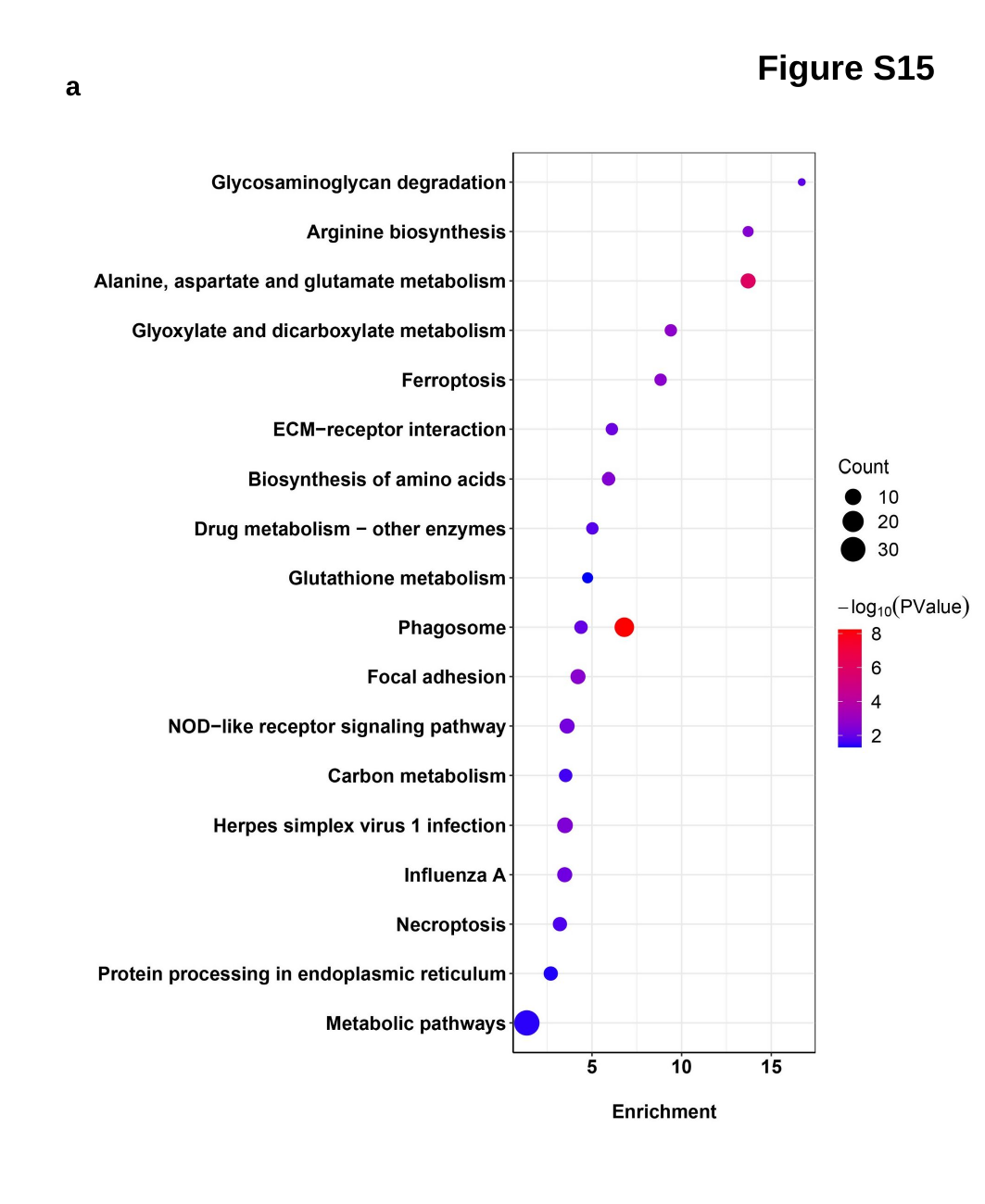

Figure S15
a

## Slide 20
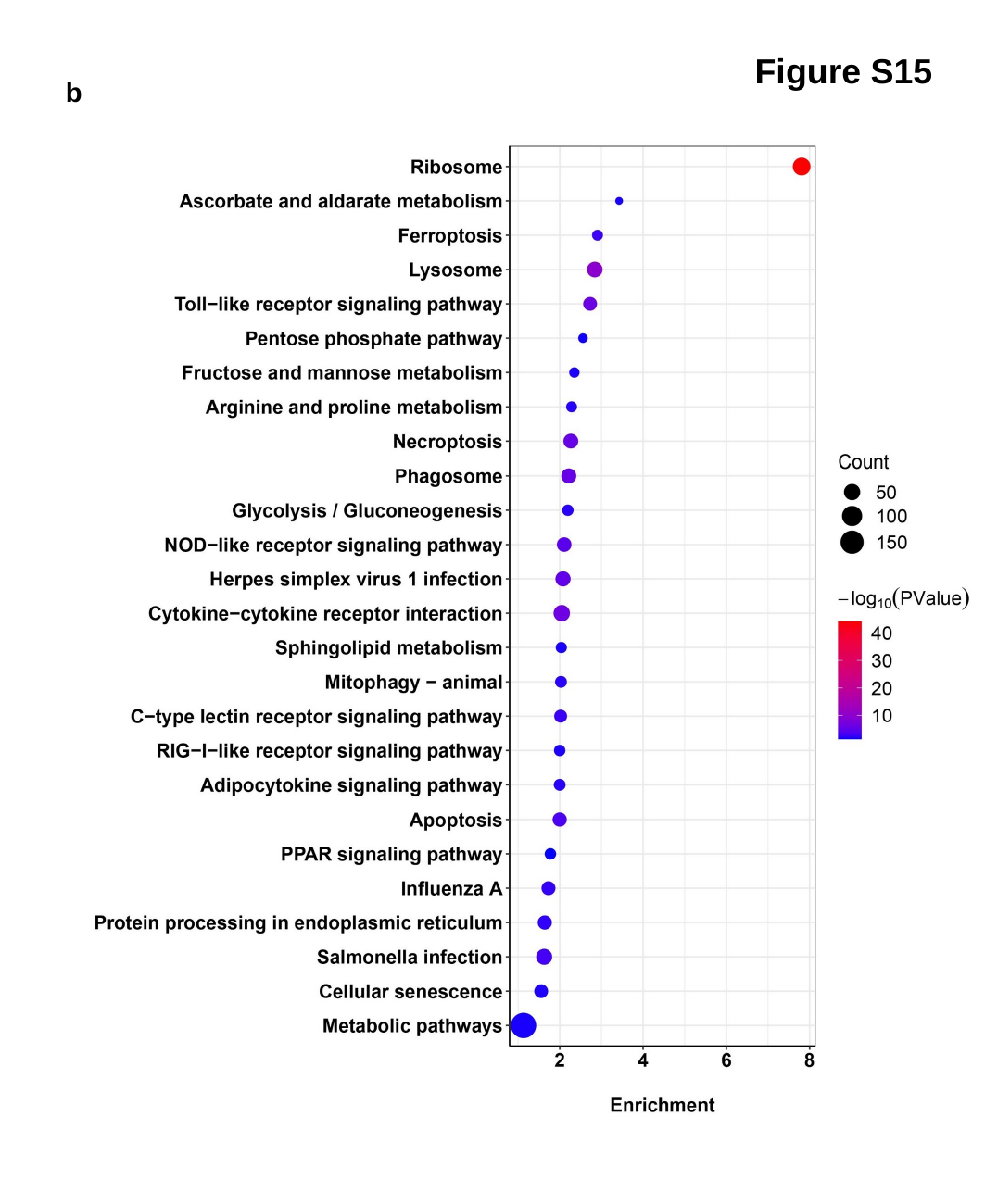

Figure S15
b

## Slide 21
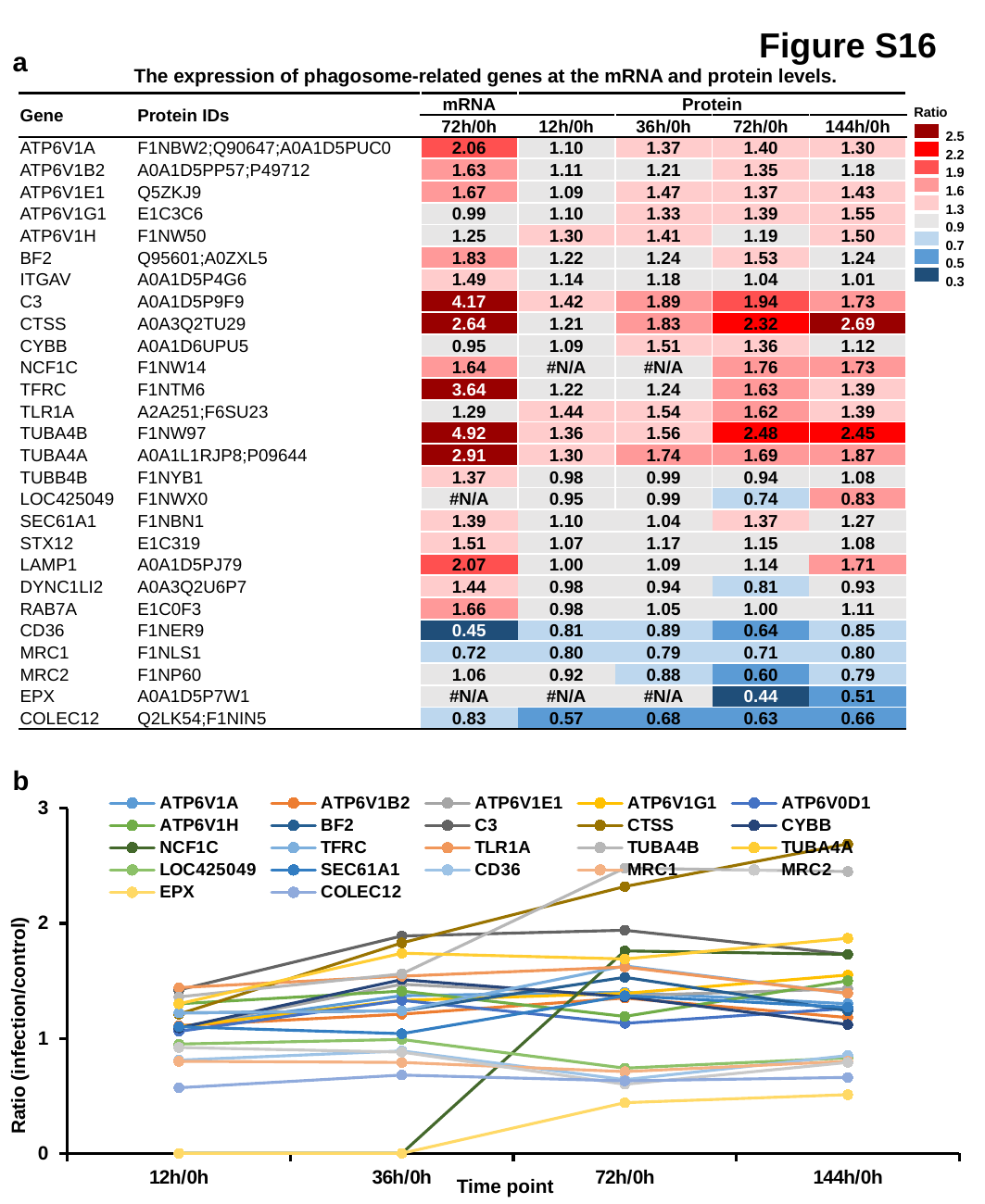

Figure S16
a
The expression of phagosome-related genes at the mRNA and protein levels.
| Gene | Protein IDs | mRNA | Protein | 36 h/ 0h | 72 h/ 0h | 144 h/ 0h |
| --- | --- | --- | --- | --- | --- | --- |
| | | 72h/0h | 12h/0h | 36h/0h | 72h/0h | 144h/0h |
| ATP6V1A | F1NBW2;Q90647;A0A1D5PUC0 | 2.06 | 1.10 | 1.37 | 1.40 | 1.30 |
| ATP6V1B2 | A0A1D5PP57;P49712 | 1.63 | 1.11 | 1.21 | 1.35 | 1.18 |
| ATP6V1E1 | Q5ZKJ9 | 1.67 | 1.09 | 1.47 | 1.37 | 1.43 |
| ATP6V1G1 | E1C3C6 | 0.99 | 1.10 | 1.33 | 1.39 | 1.55 |
| ATP6V1H | F1NW50 | 1.25 | 1.30 | 1.41 | 1.19 | 1.50 |
| BF2 | Q95601;A0ZXL5 | 1.83 | 1.22 | 1.24 | 1.53 | 1.24 |
| ITGAV | A0A1D5P4G6 | 1.49 | 1.14 | 1.18 | 1.04 | 1.01 |
| C3 | A0A1D5P9F9 | 4.17 | 1.42 | 1.89 | 1.94 | 1.73 |
| CTSS | A0A3Q2TU29 | 2.64 | 1.21 | 1.83 | 2.32 | 2.69 |
| CYBB | A0A1D6UPU5 | 0.95 | 1.09 | 1.51 | 1.36 | 1.12 |
| NCF1C | F1NW14 | 1.64 | #N/A | #N/A | 1.76 | 1.73 |
| TFRC | F1NTM6 | 3.64 | 1.22 | 1.24 | 1.63 | 1.39 |
| TLR1A | A2A251;F6SU23 | 1.29 | 1.44 | 1.54 | 1.62 | 1.39 |
| TUBA4B | F1NW97 | 4.92 | 1.36 | 1.56 | 2.48 | 2.45 |
| TUBA4A | A0A1L1RJP8;P09644 | 2.91 | 1.30 | 1.74 | 1.69 | 1.87 |
| TUBB4B | F1NYB1 | 1.37 | 0.98 | 0.99 | 0.94 | 1.08 |
| LOC425049 | F1NWX0 | #N/A | 0.95 | 0.99 | 0.74 | 0.83 |
| SEC61A1 | F1NBN1 | 1.39 | 1.10 | 1.04 | 1.37 | 1.27 |
| STX12 | E1C319 | 1.51 | 1.07 | 1.17 | 1.15 | 1.08 |
| LAMP1 | A0A1D5PJ79 | 2.07 | 1.00 | 1.09 | 1.14 | 1.71 |
| DYNC1LI2 | A0A3Q2U6P7 | 1.44 | 0.98 | 0.94 | 0.81 | 0.93 |
| RAB7A | E1C0F3 | 1.66 | 0.98 | 1.05 | 1.00 | 1.11 |
| CD36 | F1NER9 | 0.45 | 0.81 | 0.89 | 0.64 | 0.85 |
| MRC1 | F1NLS1 | 0.72 | 0.80 | 0.79 | 0.71 | 0.80 |
| MRC2 | F1NP60 | 1.06 | 0.92 | 0.88 | 0.60 | 0.79 |
| EPX | A0A1D5P7W1 | #N/A | #N/A | #N/A | 0.44 | 0.51 |
| COLEC12 | Q2LK54;F1NIN5 | 0.83 | 0.57 | 0.68 | 0.63 | 0.66 |
Ratio
2.5
2.2
1.9
1.6
1.3
0.9
0.7
0.5
0.3
### Chart
| Category | ATP6V1A | ATP6V1B2 | ATP6V1E1 | ATP6V1G1 | ATP6V0D1 | ATP6V1H | BF2 | C3 | CTSS  | CYBB | NCF1C | TFRC | TLR1A | TUBA4B | TUBA4A | LOC425049 | SEC61A1 | CD36 | MRC1 | MRC2 | EPX | COLEC12 |
|---|---|---|---|---|---|---|---|---|---|---|---|---|---|---|---|---|---|---|---|---|---|---|
| 12h/0h | 1.1 | 1.11 | 1.09 | 1.1 | 1.06 | 1.3 | 1.22 | 1.42 | 1.21 | 1.09 | 0.0 | 1.22 | 1.44 | 1.36 | 1.3 | 0.95 | 1.1 | 0.81 | 0.8 | 0.92 | 0.0 | 0.57 |
| 36h/0h | 1.37 | 1.21 | 1.47 | 1.33 | 1.33 | 1.41 | 1.24 | 1.89 | 1.83 | 1.51 | 0.0 | 1.24 | 1.54 | 1.56 | 1.74 | 0.99 | 1.04 | 0.89 | 0.79 | 0.88 | 0.0 | 0.68 |
| 72h/0h | 1.4 | 1.35 | 1.37 | 1.39 | 1.13 | 1.19 | 1.53 | 1.94 | 2.32 | 1.36 | 1.76 | 1.63 | 1.62 | 2.48 | 1.69 | 0.74 | 1.37 | 0.64 | 0.71 | 0.6 | 0.44 | 0.63 |
| 144h/0h | 1.3 | 1.18 | 1.43 | 1.55 | 1.26 | 1.5 | 1.24 | 1.73 | 2.69 | 1.12 | 1.73 | 1.39 | 1.39 | 2.45 | 1.87 | 0.83 | 1.27 | 0.85 | 0.8 | 0.79 | 0.51 | 0.66 |b
Ratio (infection/control)
Time point

## Slide 22
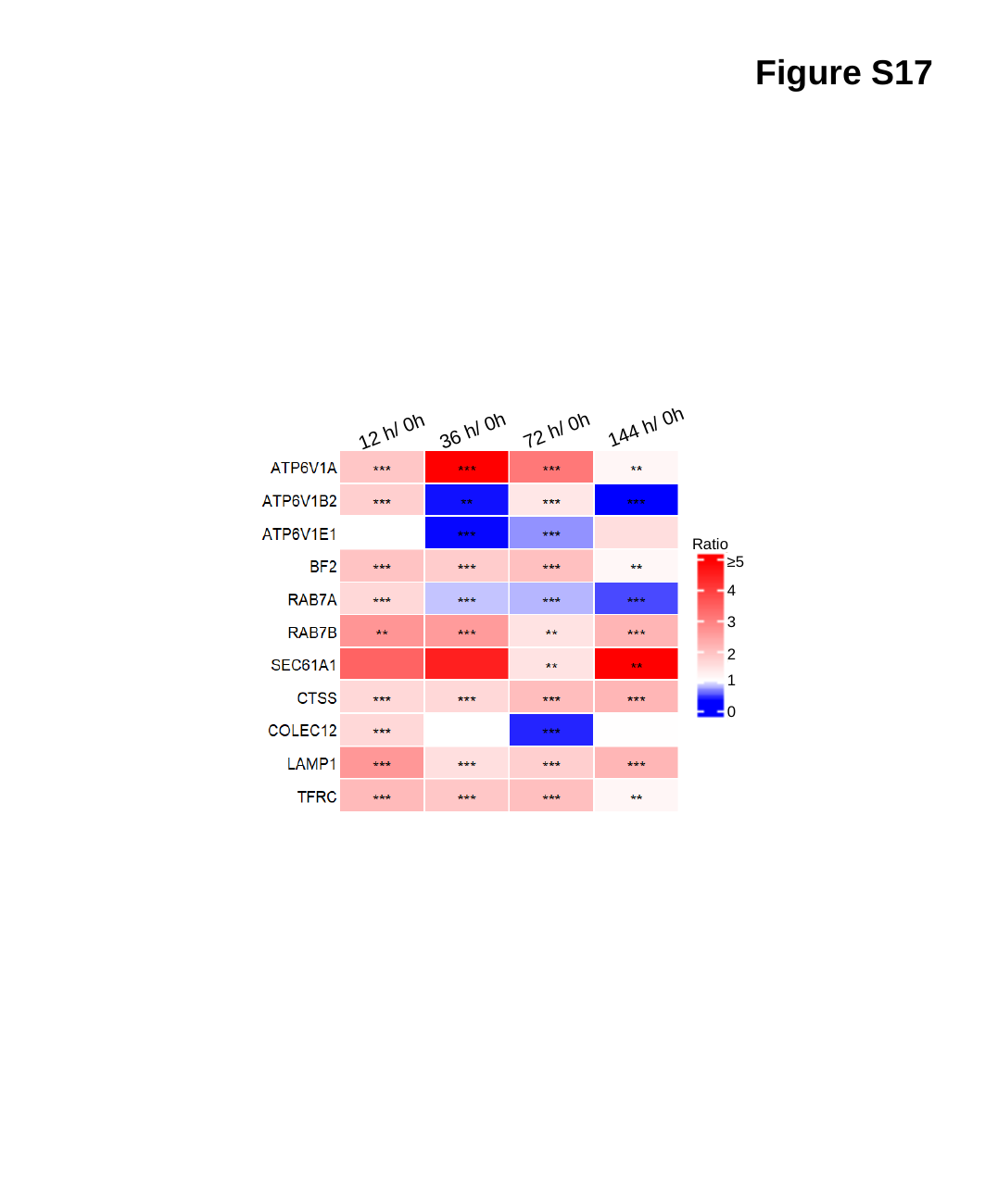

Figure S17
144 h/ 0h
72 h/ 0h
36 h/ 0h
12 h/ 0h
Ratio
≥5
4
3
2
1
0
